# Long-term safety and maintenance of response with esketamine nasal spray in participants with treatment-resistant depression: interim results of the SUSTAIN-3 study

**DOI:** 10.1038/s41386-023-01577-5

**Published:** 2023-05-12

**Authors:** Naim Zaki, Li (Nancy) Chen, Rosanne Lane, Teodora Doherty, Wayne C. Drevets, Randall L. Morrison, Gerard Sanacora, Samuel T. Wilkinson, Vanina Popova, Dong-Jing Fu

**Affiliations:** 1 Department of Neuroscience, Janssen Research & Development, LLC, Titusville, NJ USA; 2 Department of Clinical Biostatistics, Janssen Research & Development, LLC, Titusville, NJ USA; 3 Department of Neuroscience, Janssen Research & Development, LLC, San Diego, CA USA; 4 Department of Psychiatry, Yale University School of Medicine, New Haven, CT USA; 5 Department of Neuroscience, Janssen Research & Development Belgium, Beerse, Belgium

**Keywords:** Medical research, Diseases

## Abstract

Patients with treatment-resistant depression (TRD) have higher rates of relapse and pronounced decreases in daily functioning and health-related quality of life compared to patients with major depressive disorder who are not treatment-resistant, underscoring the need for treatment choices with sustained efficacy and long-term tolerability. Adults with TRD who participated in ≥1 of 6 phase 3 “parent” studies could continue esketamine treatment, combined with an oral antidepressant, by enrolling in phase 3, open-label, long-term extension study, SUSTAIN-3. Based on their status at parent-study end, eligible participants entered a 4-week induction phase followed by an optimization/maintenance phase, or directly entered the optimization/maintenance phase of SUSTAIN-3. Intranasal esketamine dosing was flexible, twice-weekly during induction and individualized to depression severity during optimization/maintenance. At the interim data cutoff (01 December 2020), 1148 participants were enrolled, 458 at induction and 690 at optimization/maintenance. Mean (median) cumulative duration of maintenance esketamine treatment was 31.5 (37.7) months (totaling 2769 cumulative patient-years). Common treatment-emergent adverse events (≥20%) were headache, dizziness, nausea, dissociation, somnolence, and nasopharyngitis. Mean Montgomery–Åsberg Depression Rating Scale (MADRS) total score decreased during induction, and this reduction persisted during optimization/maintenance (mean [SD] change from the baseline to the endpoint of each phase: induction −12.8 [9.73]; optimization/maintenance +1.1 [9.93]), with 35.6% and 46.1% of participants in remission (MADRS total score ≤12) at induction and optimization/maintenance endpoints, respectively. Improvement in depression ratings generally persisted among participants who remained in maintenance treatment, and no new safety signal was identified during long-term treatment (up to 4.5 years) using intermittent-dosed esketamine in conjunction with daily antidepressant.

## Introduction

Approximately one-third of patients with major depressive disorder (MDD) do not achieve an antidepressant response despite treatment with multiple antidepressants and are considered to have treatment-resistant depression (TRD) [1, 2]. Patients with TRD have higher rates of relapse and higher suicide rates, compared to patients with MDD who are not treatment resistant [3, 4]. Even when patients with TRD respond, the relapse rate is high (~70%) within 6 months [5]. Patients with TRD have pronounced decreases in daily functioning and health-related quality of life, compared to non-TRD MDD patients [6], underscoring the need for treatment choices with sustained efficacy and tolerability over the long-term.

Esketamine nasal spray (Spravato^®,^ Janssen Pharmaceuticals Inc., Titusville, NJ), in conjunction with a newly-initiated oral antidepressant, has been approved for TRD by the US Food and Drug Administration, the European Medicines Agency [7, 8], and other health authorities in over 70 countries. The approvals of esketamine nasal spray were based on efficacy and safety findings from phase 2/3 studies of TRD treatment for 4 weeks to 1 year [9–14]. SUSTAIN-3 is assessing the long-term safety and efficacy of individualized, intermittently-dosed esketamine nasal spray, in conjunction with an oral antidepressant, in patients with TRD. Given clinical interest for long-term safety and efficacy data with esketamine, results of an interim analysis of SUSTAIN-3 data, based on a database lock on 01 December 2020, are reported herein.

## Methods

### Ethical practices

An Institutional Review Board (United States) or Independent Ethics Committee (all other locations) approved the study protocol and its amendments. The study is being conducted in accordance with ethical principles of the Declaration of Helsinki, Good Clinical Practices (GCP), and applicable regulatory requirements. Written consent was obtained from all participants prior to enrollment.

### Study design

SUSTAIN-3, an ongoing phase 3, open-label, multicenter (*n* = 59), long-term extension study, was initiated in June 2016; the last participant was enrolled in February 2019. The study will complete in December 2022.

SUSTAIN-3 has a 4-week induction phase (if applicable) and an optimization/maintenance phase of variable duration. Participants in 1 of 6 phase 3, “parent” studies of esketamine and for whom benefit versus risk was favorable were enrolled into either the 4-week induction phase or the long-term optimization/maintenance phase of SUSTAIN-3 based on their status at the end of the parent study (Fig. S1; Table S1).

SUSTAIN-3 (clinical trials.gov identifier: NCT02782104) and the parent studies (identifiers: NCT02417064, NCT02418585, NCT02422186, NCT02493868, NCT02497287, NCT03434041) are registered at clinicaltrials.gov.

### Study population

The eligibility criteria of each parent study are reported elsewhere [10–14; clinical trials.gov: ID NCT03434041]. In brief, each parent study enrolled adults (≥18 years) who met the definition of TRD (i.e., non-response to an adequate trial of at least 2 antidepressants in the current episode of depression, one of which was observed prospectively).

### Study drug

In the induction phase, participants self-administered (under medical supervision) esketamine (28 mg [starting dose age ≥65 years], 56 mg, or 84 mg) as a flexible dose, twice-weekly therapy for 4 weeks. In the optimization/maintenance phase, participants received interval dosing of esketamine individualized to the severity of their depression based upon a clinical global impression - severity [CGI-S]-based algorithm (refer to Table S2). Throughout the study participants also were prescribed an oral antidepressant determined by the investigator, except monoamine oxidase inhibitor.

Throughout the study, esketamine was dispensed only at clinical sites during each dosing session and dose administration occurred under direct supervision of site staff.

### Evaluations of safety and efficacy

#### Safety

Treatment-emergent adverse events, including events of special interest (i.e., renal disorder/interstitial cystitis, hepatic) were monitored, and other safety assessments (i.e., hematology and serum chemistry, urinalysis, physical examination, pulse oximetry, vital signs, electrocardiogram, and Columbia-Suicide Severity Rating Scale [15] [C-SSRS] to assess potential suicidal ideation and behavior) were performed throughout the study. Urine drug screening for illicit drugs (e.g., cocaine, methadone, opiate, stimulants) was performed every 12 weeks. Urinalysis was performed at baseline, day 28, and then quarterly. For adverse events related to urinary symptoms, investigators were queried for the etiology/risk factors for the event, results of urine culture if performed, and follow-up from referral to a urologist/gynecologist when necessary.

Modified Observer’s Assessment of Alertness/Sedation (MOAA/S) scale was used to assess the level of post-dose sedation, and the Clinical Global Assessment of Discharge Readiness (CGADR), to assess participants’ discharge readiness, based on their overall clinical status.

The Cogstate computerized test battery [16, 17] was used to assess multiple cognitive domains, including attention (simple and choice reaction time), visual learning and memory, and executive function; the Hopkins Verbal Learning Test-Revised [18] (HVLT-R) was used to measure verbal learning and memory.

#### Efficacy

Montgomery–Åsberg Depression Rating Scale (MADRS) assessments [19] were performed at baseline and weekly in the induction phase and every 8 weeks thereafter.

Participants rated the impact of the study treatments on socio-occupational disability using the Sheehan Disability Scale [20] (SDS) and on depressive symptoms using the Patient Health Questionnaire 9-item [21] (PHQ-9).

Investigators rated severity of depressive illness using the CGI-S [22], which was also used to determine treatment session frequency in the optimization/maintenance phase.

### Statistical methods

The number (percentage) of participants with adverse events, including events of clinical interest, serious adverse events, and adverse events leading to premature discontinuation of study drug were summarized by preferred term. Descriptive statistics were provided for other safety parameters.

Descriptive statistics and frequency distributions were used to summarize the efficacy data. Efficacy endpoints for both the induction and optimization/maintenance phases comprise the following: change from baseline in depressive symptoms (MADRS and PHQ-9), including response (≥50% improvement from baseline) and remission (MADRS total score ≤ 12; PHQ-9 total score < 5 [23]); overall severity of illness (CGI-S); and functioning and associated disability (change from baseline in SDS; response defined as SDS scores ≤4 for each item and ≤12 for the score; remission defined as SDS ≤ 2 for each item score and ≤6 for the total score [24]).

## Results

**Fig. 1:**
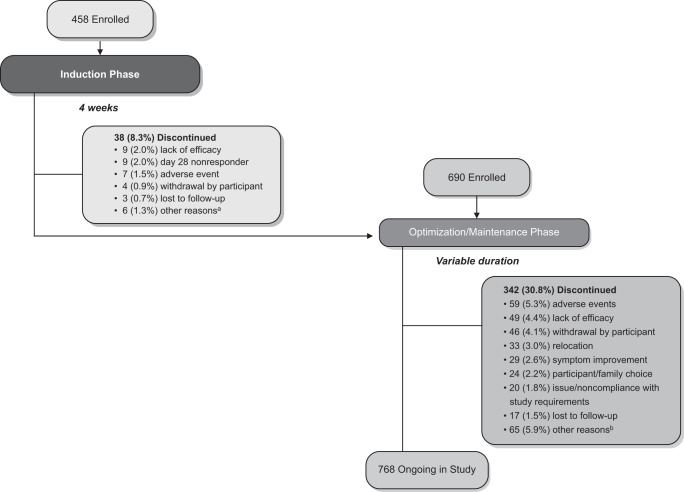
SUSTAIN-3 Participant Disposition. ^a^One each: scheduling conflicts; multiple reasons (mainly, feeling better, wanted to start working again, adverse events, and time requirements of study); withdrew consent; participant’s choice despite investigator’s advice to continue; relocation; and death on study day 26 due to completed suicide, 4 days after the last dose of esketamine. ^b^Other reasons (each ≤1%, e.g., investigator/sponsor decision, employment/school, personal reasons, death [4 participants, as described in Supplementary Material]). Note: Participants were eligible to enroll into the Induction Phase or the Optimization/Maintenance Phase based on their status at the end of the parent study (refer to Table S2). Participants received open-label esketamine nasal spray (28 mg [only an option for participants ≥65 years], 56 mg, or 84 mg) twice per week during the Induction Phase, and weekly, every other week, or every 4 weeks, based on clinical global impression - severity (CGI-S) and tolerability, during the Optimization/Maintenance Phase.

A total of 1148 adult patients with TRD were enrolled into SUSTAIN-3. Overall, 458 participants were enrolled into the induction phase, 38 (8.3%) of whom discontinued and 420 (91.7%) continued to the optimization/maintenance phase. In addition, 690 other participants were enrolled directly into the optimization/maintenance phase (Fig. 1). Of 1110 patients who participated in the optimization/maintenance phase, 342 (30.8%) discontinued the study for various reasons. The reasons for treatment discontinuation are provided in Fig. 1 and include adverse events (*n* = 59, 5.3%), lack of efficacy (*n* = 49, 4.4%), and sustained symptom improvement (*n* = 29, 2.6%). No clear trend was evident in the frequency of discontinuations across time based on the length of time in study (0 - ≤ 6 months: 98 participants [8.8%]; 6 months - ≤ 1 year: 65 participants [5.9%]; 1 year - ≤ 2 years: 98 participants [8.8%]; ≥2 years: 81 participants [7.3%]). At the interim database lock (01 December 2020), 768 participants were ongoing in the study (Fig. 1).

Demographic characteristics of the study cohort are shown in Table 1.

**Table 1:** Demographic and clinical characteristics at baseline.

	Esketamine Nasal Spray *N* = 1148^a^
Age (years)	
Mean (SD)	49.6 (12.28)
≥ 65	122 (10.6)
Sex, *n* (%)	
Male	384 (33.4)
Female	764 (66.6)
Race, *n* (%)	
Asian	45 (3.9)
Black or African American	45 (3.9)
White	996 (86.8)
Other	61 (5.3)
Employment status^b^, *n* (%)	
Any type of employment	693 (60.4)
Any type of unemployment	281 (24.5)
Other	174 (15.2)
History of hypertension prior to study participation, *n* (%)	
Yes	271 (23.6)
No	877 (76.4)
Region, *n* (%)	
Europe	486 (42.3)
North America	343 (29.9)
South America	217 (18.9)
Africa	48 (4.2)
Asia	38 (3.3)
Oceania	16 1.4)

^a^*N* = 1147 for race.

^b^Any type of employment includes: any category containing “Employed” (full-time, part-time), Sick or Disability Leave, Sheltered Work, Housewife or Dependent Husband, and Student; any type of unemployment includes: any category containing “Unemployed”; Other includes: Retired and No Information Available.

At the interim database lock, mean exposure to esketamine nasal spray in SUSTAIN-3 was 31.5 months (median 37.7, range 0–56 months), with 930 (81.0%) participants exposed for ≥12 months, 830 (72.3%) for ≥24 months, 556 (48.4%) for ≥36 months, and 6 (0.5%) for ≥48 months; total exposure was 2769 cumulative patient-years. The mean (range) cumulative duration of intermittent esketamine treatment during the parent and SUSTAIN-3 studies combined was 36.8 (0–64) months, with 991 (86.3%) participants treated for ≥12 months, 866 (75.4%) for ≥24 months, 726 (63.2%) for ≥36 months, and 173 (15.1%) for ≥48 months; total exposure was 3238 cumulative patient-years.

Most participants received the maximum 84 mg esketamine dose (66.4%), and fewer received the 56 mg dose (31.0%), as their most recent dose before the interim data cutoff. Most participants received esketamine either weekly or biweekly (every other week) during their first, second, and third year of participation in SUSTAIN-3 (Table S3).

The most common concomitant oral antidepressants were duloxetine, venlafaxine, escitalopram, and sertraline (Table S4).

### Safety

#### Treatment-emergent adverse events

The five most common adverse events were dissociation, dizziness, nausea, vertigo, and dysgeusia during the induction phase and headache, dizziness, nausea, dissociation, and nasopharyngitis in the optimization/maintenance phase (Table 2). No events of psychosis were reported. Most (96.8%) adverse events were mild or moderate in severity. Among participants having severe events, the most common were dysgeusia (2.6%), dissociation (2.2%), and dizziness (2.0%) during the induction phase and dysgeusia (2.3%) during the optimization/maintenance phase. All severe events of dysgeusia occurred and resolved on the day of dosing. Severe events of dissociation occurred only in the induction phase and generally resolved within 90 min. Adverse events comprising seeking, overdose, or abuse of esketamine (or ketamine) were not reported by the study site clinicians.

**Table 2:** Most frequently reported adverse events^a^.

	Esketamine Nasal Spray
Induction Phase (*N* = 458)	
Dissociation	100 (21.8%)
Dizziness	94 (20.5%)
Nausea	81 (17.7%)
Vertigo	77 (16.8%)
Dysgeusia (bad/altered taste)	76 (16.6%)
Headache	69 (15.1%)
Optimization/Maintenance Phase (*N* = 1110)	
Headache	369 (33.2%)
Dizziness	342 (30.8%)
Nausea	332 (29.9%)
Dissociation	257 (23.2%)
Nasopharyngitis	251 (22.6%)
Somnolence	246 (22.2%)
Dysgeusia	208 (18.7%)
Vertigo	196 (17.7%)
Back pain	189 (17.0%)
Anxiety	175 (15.8%)
Vomiting	161 (14.5%)
Diarrhea	155 (14.0%)
Urinary tract infection	148 (13.3%)
Blood pressure increased	141 (12.7%)
Upper respiratory tract infection	131 (11.8%)
Insomnia	122 (11.0%)
Influenza	115 (10.4%)
Vision blurred	114 (10.3%)

^a^≥10% of participants.

Serious adverse events were reported for 171 (14.9%) of the 1148 participants during SUSTAIN-3, over 2769 cumulative patient-years. The most common serious adverse event reported (1.5% of participants) was (worsening) depression; all other serious events were reported for <1% of participants (Table S5). Investigators considered the majority (98.5%) of serious adverse events to be doubtfully or not related to esketamine. There were 5 (0.4%) deaths, none considered by the investigator as related to esketamine (details in Supplementary Material).

Of the 1148 participants entered, 67 (5.8%) discontinued study drug due to adverse events. The most common adverse events (>1 participant) leading to discontinuation of esketamine were: (worsening) depression or major depression (7 [0.6%]), blood pressure increased (6 [0.5%]), dissociation (5 [0.4%]), anxiety (3 [0.3%]), mania (3 [0.3%]), fatigue (2 [0.2%]), and suicidal ideation (2 [0.2%]).

#### Dissociation

Dissociation included reports of perceptual disturbances where sounds, visual stimuli, and proprioception seemed exaggerated or altered, or by a sense of derealization or depersonalization. Overall, dissociation was reported in 24.4% of participants. Although no treatment for dissociation was recommended or specified in the study protocol, a few participants (0.8%) received treatment with concomitant medication(s) for dissociation, which primarily (4 of 8 participants) consisted of single doses of a benzodiazepine anxiolytic (alprazolam, diazepam, lorazepam). There were no serious adverse events of dissociation.

Almost all (5358/5369, 99.8%) adverse events of dissociation occurred and resolved on the day of dosing. Seven (0.6%) participants had 1 or more dissociation events that resolved after the day of dosing, all within 2 days after dosing. No participant had esketamine dose changed due to dissociation.

#### Sedation

Sedation was reported as an adverse event in 7.8% of participants. The majority (99.4%, 1073/1079) of sedation events occurred on a dosing day and resolved the same day. Five (0.4%) participants had 1 or more sedation events that did not resolve on the day of dosing. Sedation led to esketamine dose reduction for 1 of these participants.

There were no serious adverse events of sedation or adverse events of sedation that led to withdrawal of study drug. No participant reported respiratory depression. An adverse event of oxygen saturation decreased was reported for four participants; all such events were transient and self-limited, none requiring any intervention. The four participants had MOAA/S scores of 5 (i.e., defined as participant readily responding to name spoken in normal tone [awake]).

The MOAA/S scores of the study participants are summarized in the Supplement. Clinically-relevant sedation, defined by MOAA/S score ≤ 3 (i.e., moderate or greater sedation), occurred at least once among 6.1% (28/458) of participants in the induction phase and in 6.9% (77/1110) of participants in the optimization/maintenance phase.

#### Increased blood pressure

The greatest mean (SD) change in systolic and diastolic blood pressure (BP) from predose values were +9.3 (12.01) mmHg at day 15 and +6.1 (7.84) mmHg at day 25, respectively, in the induction phase and +10.2 (9.25) mmHg at week 184 and +6.0 (6.36) mmHg at week 184, respectively, in the optimization/maintenance phase, all at 40 min post-dose. Participants who met the study criteria for markedly elevated BP (i.e., systolic BP ≥ 180 mmHg or diastolic BP ≥ 110 mmHg) are summarized in the Supplement.

Overall, investigators reported an adverse event related to increased BP for 17.9% of participants, with incidence generally similar at visits throughout both the induction and optimization/maintenance phases (Fig. S2). Most (≥96%) increased BP events occurred and resolved on the day of dosing.

#### Adverse events of clinical interest

No case of treatment-related interstitial/ulcerative cystitis was identified. Urinary tract infections (UTI) were reported in 153 (13.3%) participants, 65 of whom had more than one episode of UTI (63 of 65 were female, mean age 53.7 years). Other adverse events (incidence ≥1%) related to a renal disorder included dysuria (2.7%), pollakiuria (2.4%), micturition urgency (1.3%), nephrolithiasis (1.3%), hematuria (1.0%), and urinary incontinence (1.0%).

A minority (6.3%) of participants experienced 1 or more hepatic adverse events, the most common being gamma glutamyl transferase increased (2.1%), alanine aminotransferase (ALT) increased (1.4%), aspartate aminotransferase increased (1.0%), hepatic enzyme increased (1.0%), and cholelithiasis (1.0%). No participant manifested hepatic enzyme levels of ALT > 3 x upper limit of normal [ULN] and total bilirubin >2 x ULN. Nine hepatic events were considered serious, including 8 events of cholelithiasis and 1 event of cholecystitis, all classified by the site investigator as not related to esketamine.

#### Discharge readiness

At the timepoints assessed, >89% of participants were ready to be discharged from clinic by 1.5 h post dosing.

#### Cognitive effects

Group mean performance on cognitive tests (Cogstate and HVLTR) from baseline through week 160 indicated that cognitive performance generally remained stable. There was no evidence of decline in cognition associated with long-term treatment among participants <65 years old from baseline to week 160 (Table S6). On tests of higher cognitive function (visual learning, working memory, executive function, verbal learning, delayed verbal memory, recognition memory), within-group analyses of mean change from baseline at endpoint for z-scores range from 0.114 to 0.277 (i.e., reflecting numerical improvement); within-group analyses of mean change from baseline for z-scores across timepoints from weeks 112–160 range from 0.090 to 0.364 (also reflecting numerical improvement). There was slight slowing of Reaction Time (RT: Cogstate Detection and Identification are simple and choice RT tests, respectively) among participants <65 years old (within group analyses of RT mean change from baseline at endpoint for z-scores: simple RT = −0.054, choice RT = −0.220); within-group analyses of RT mean changes from baseline for z-scores range from −0.072 to 0.058 for simple RT and −0.270 to −0.095 for choice RT across timepoints weeks 112–160. Participants ≥65 years of age evidenced somewhat greater slowing of both simple and choice RT relative to baseline (within-group mean change from baseline at endpoint for z-scores: simple RT = −0.195; choice RT = −0.368) (Table S7). Slowing first appeared early in the optimization/maintenance phase, became more apparent at approximately week 40, and worsened through approximately week 88, after which mean RT scores began to stabilize (within-group analyses of mean RT changes from baseline for z-scores range: −0.441 to 0.089 for simple RT and −0.685 to -0.519 for choice RT across timepoints weeks 112–160; *n* = 62 at baseline and week 160) (Figs. S3, S4). Beyond week 160 the sample size was insufficient for interpretation. There was considerable intraindividual variability (IIV) in RT trajectories (i.e., for a considerable number of participants, RTs fluctuated across assessment timepoints, as opposed to steadily declining). IIV likely contributed to variability of group means over time, as represented in variable mean changes from baseline across timepoints. Among the participants ≥65 years old, there was no evidence of decline on any measure of higher cognitive function. Performance on all tests of higher cognitive function remained stable or slightly improved (within-group mean change from baseline at endpoint for z-scores ranging from 0.022 to 0.306; at weeks 112–160, within-group mean change from baseline for z-scores ranging from −0.094 to 0.583).

#### Suicidal ideation and behavior

Overall, 5.6% of participants experienced 1 or more adverse events potentially related to suicidality. Of these, one participant died by suicide. This individual was a 48-year-old male who did not respond to esketamine (MADRS total score was 35 at baseline and 41 and 25 on days 15 and 22, respectively). The participant died by suicide 4 days after the most recent esketamine dose. Considering this individual’s long history of mental illness and underlying TRD, the event was reported by the site investigator as not related to esketamine.

In the C-SSRS assessment, 49 participants (of 1144; 4.3%) with no history of suicidal ideation reported new occurrences of suicidal ideation during the study. Ten (0.9%) participants reported new suicidal behavior, 9 of whom had a previous history of suicidal ideation. Improvement in severity category of C-SSRS assessment from baseline to postbaseline occurred in 14.0% of participants (*n* = 160) (Table S8).

### Efficacy

#### Depressive symptoms

**Fig. 2:**
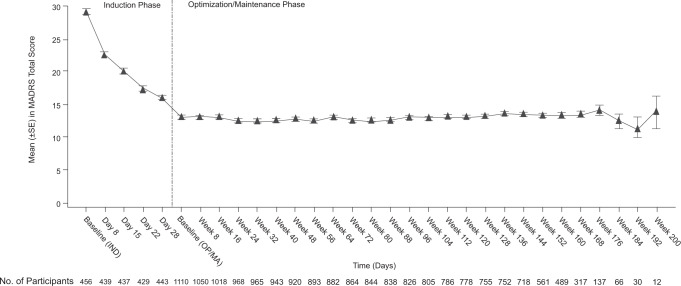
Mean (±SE) Montgomery–Åsberg Depression Rating Scale Total Score (Observed Cases). IND Induction, OP/MA optimization/Maintenance. Note: Responders from the induction phase of the SUSTAIN-3 study and responders from parent studies were to enter the optimization/maintenance phase. The greater variability of the mean MADRS total score at later time points likely reflects the smaller number of participants at these timepoints, as reflected in the corresponding sample sizes.

Depressive symptoms, as assessed by MADRS total score, decreased during the induction phase (Fig. 2); the improvement was maintained during the optimization/maintenance phase (mean [SD], 95% CI change from the baseline to the endpoint of each phase: induction, −12.8 [9.73], −13.66 to −11.87; optimization/maintenance, +1.1 [9.93], 0.48 to 1.65).

The proportion of responders (defined as ≥50% reduction in MADRS total score; observed cases) increased over time during the induction phase, from 15.0% (66/439) on day 8 to 50.6% (224/443) on day 28 and to 49.2% (224/455) at endpoint of the induction phase. A total of 35.6% (162/455) of participants were in remission (MADRS total score ≤12; observed cases) at the induction endpoint, and 50.9% (464/911) and 46.1% (511/1108) at year 1 and endpoint of the optimization/maintenance phase, respectively.

Improvement in depressive symptoms was also noted based on decrease in PHQ-9 total score over the course of the induction phase (mean [SD] change from baseline to phase endpoint: −5.8 [5.84]; 95% CI: −6.32 to −5.24), with improvement maintained during the optimization/maintenance phase (change from phase baseline to endpoint: +0.9 [6.04]; 95% CI: 0.50 to 1.21) (Fig. S5). The percentage of participants who characterized their depressive symptoms as moderately severe to severe, based on PHQ-9 score, decreased from baseline (56.5%, 258/456) to endpoint of the induction phase (20.4%, 93/454) and endpoint of the optimization/maintenance phase (18.1%, 201/1108). The percentage of responders (defined as ≥50% improvement in PHQ-9 total score) was 37.6% (170/452) at endpoint of the induction phase. The percentage of remitters (defined as a PHQ-9 total score <5) was 19.8% (90/454) and 31.9% (354/1108) at endpoint of the induction and optimization/maintenance phases, respectively.

**Fig. 3:**
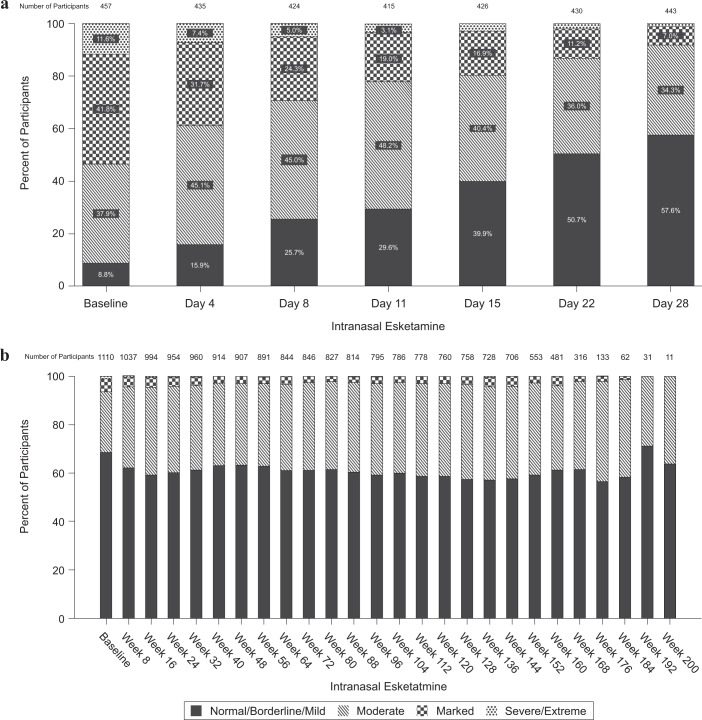
Clinical global impression–severity (CGI-S): frequency distribution over time. **a** Induction Phase. **b** Optimization/Maintenance Phase. Notes: Every-8-week data are presented. The visits with fewer than 10 participants are not presented. The CGI-S evaluates the severity of psychopathology on a scale of 1 to 7: 1 = normal (not at all ill); 2 = borderline mentally ill; 3 = mildly ill; 4 = moderately ill; 5 = markedly ill; 6 = severely ill; 7 = among the most extremely ill patients.

Illness severity, as assessed by the CGI-S, improved from baseline (median of 5) to the endpoint of the induction phase (median [range] change from baseline, −1.0 [−5; 1]) and remained stable over the optimization/maintenance phase (median [range] change: 0.0 [−5; 4]), suggesting maintenance of the antidepressant effect. More than half of participants had CGI-S scores indicating normal/borderline/mild illness (scores of 1, 2, or 3) at endpoint of the induction phase (55.9%) and at endpoint of the optimization/maintenance phase (57.3%) (Fig. 3).

#### Functioning and associated disability

The mean change (SD) from baseline to endpoint in SDS total score of −6.4 (7.14) indicates an improvement in functioning and associated disability during the induction phase. By endpoint of the optimization/maintenance phase, the mean change (SD) was +0.1 (8.23), suggesting maintenance of effect. The percentage of responders (defined as SDS scores ≤4 for each item and ≤12 for the total score) was 44.8% and 54.4% at endpoint of the induction and optimization/maintenance phases, respectively, and percentage of participants in remission (defined as SDS ≤ 2 for each item score and ≤6 for the total score) (Fig. S6) was 22.9% (89/388) and 36.6% (393/1075) at the respective phase endpoints.

## Discussion

Long-term safety as well as remission and response with weekly or biweekly esketamine nasal spray, combined with an oral antidepressant, is being evaluated in SUSTAIN-3, a global multicenter study of participants with TRD. The results of this study extend those of a 1-year open-label study of esketamine nasal spray for TRD [14], addressing clinical interest in long-term safety and efficacy data [25]. At the interim analysis the mean duration of intermittent esketamine treatment in this trial was 31.5 months (range up to 56 months). The relatively high rate of 76% (849/1110) of study participants with TRD who remained in the optimization/maintenance phase of the SUSTAIN-3 study for 2 years or longer is noteworthy, given that each esketamine administration required an in-person clinic visit and that the study was conducted during the height of the COVID pandemic. Some participants discontinued because they felt better, wanted to start working again, or relocated. Crucially, the discontinuation rate due to lack of efficacy was relatively low (4.4%), as shown in Fig. 1 and discussed below within the context of pertinent literature.

The safety profile of esketamine, with continuous intermittent dosing for up to 4.5 years in SUSTAIN-3 (2,769 cumulative patient-years), is consistent with its established safety profile in participants with TRD treated for up to 1 year [7, 8, 14]. In the current study, most adverse events were not clinically significant, were mild or moderate in intensity, and were transient. Nearly all adverse events of dissociation and sedation occurred and resolved on the day of dosing. The incidence of clinically-relevant sedation (MOAA/S score ≤ 3) in SUSTAIN-3 (6.1% in the induction phase and 6.9% in the optimization/maintenance phase) is similar to that observed in the prior long-term SUSTAIN-2 study (8.4% and 7.0% in the respective phases) [14]. Psychosis was not reported. Regarding the urinary tract symptoms, there was no case of treatment-related interstitial/ulcerative cystitis, and the incidence of urinary tract infections of 13.3% in SUSTAIN-3 is comparable with a rate of 14.5% observed in a similar cohort of patients who did not receive esketamine (69.7% female; mean [SD] age, 49.2 [18.6] years) within a health claims database during the year prior to diagnosis of TRD (data on file, from Optum^®^ Clinformatics^TM^ 2021 [26]).

While ketamine abuse could not have been detected if participants in SUSTAIN-3 surreptitiously obtained drug from illicit sources (the urine drug screen we used did not assay for the r-enantiomer of ketamine or its metabolites), the protocol represents a rigorous approach to estimating the abuse liability in a clinical population. In this regard, adverse events related to abuse of esketamine (or ketamine) were not reported by the site clinicians. Moreover, diversion or excess use was putatively prevented in the clinical trial setting as only one dose could be dispensed during each dosing session and each administration of esketamine occurred under clinical supervision. Additionally, a study drug reconciliation process was implemented.

A minority of study participants discontinued esketamine treatment due to an adverse event. Likewise, few (1.5%, 4/259) serious adverse events were attributed to esketamine by the site investigators. Long-term exposure to esketamine yielded no additional concerns or trends related to suicidal ideation and/or behavior, drug abuse, or drug dependence.

While up to 20% of patients with MDD attempt suicide over their lifetime [27, 28], with an estimated lifetime risk of 3.4% for suicide death in this population [29], among patients with TRD the incidences of suicide attempts, suicide death, and all-cause mortality are higher than in the general MDD population [30, 31]. In a meta-analysis of studies assessing various treatments for TRD, the rates of non-fatal suicidal behavior and suicide death were 4.66 and 0.47 per 100 patient-years, respectively [32–34]. In the current study, 49 (4.3%) patients who had no history of suicidal ideation reported a new occurrence of suicidal ideation at some time during their participation in the study, 10 patients (0.9%; of whom 9 had a known history of suicidal ideation) attempted suicide (0.361 per 100 patient-years), and 1 died by suicide during the study period (0.036 per 100 patient-years). The rates of all-cause mortality for TRD previously reported in the clinical literature (0.79 [30] to 4.6 [31] per 100 patient-years) are higher than that observed in SUSTAIN-3 (5 deaths, 0.181 per 100 patient-years).

Cognition was assessed across multiple cognitive domains and remained stable, without changes over time, for the total sample and for the subgroup of participants <65 years of age. In participants ≥65 years, performance on all tests of higher cognitive function remained stable or slightly improved. However, among participants ≥65 years, slowing of simple and choice RT occurred during the optimization/maintenance period; mean change from baseline for z-score calculations indicated that the changes were in a range that would be characterized as a small effect. Slowing increased through approximately week 100, after which RT performance appeared to stabilize through week 160. The clinical relevance of the observed slowing of RT is unclear. Overall, attentional ability was not affected, and in the absence of a placebo group it is difficult to determine to what extent the slowing of RT reflects an effect of study drug. Slowing of RT/processing speed, possibly associated with increasing IIV of RT performance over time, has been observed in multiple longitudinal studies in older individuals, including patients with MDD [35]. In a longitudinal study of healthy volunteers, participants in their 40’s evidenced small increases in IIV on RT performance on choice reaction time tasks, but not on simple RT, whereas those in their 60’s showed more pronounced increases in both IIV and RT on simple and choice RT tasks [36].

On average, the participants showed improvement in measures of depressive symptoms and other efficacy assessments during the induction phase (first 4 weeks of exposure), which appeared to be sustained during the optimization/maintenance phase. At the interim data cutoff, the majority of participants were receiving the 84 mg or 56 mg esketamine dose, either weekly or every other week, to maintain clinical stability. In the remainder, the every-4-week dosing interval was used only by about 14% of participants at the 1 year mark and close to 17% of participants at the 2 year mark, as shown in Table S3. The interim efficacy results from SUSTAIN-3 are consistent with the results of a trial testing the maintenance efficacy of esketamine plus oral antidepressant using a randomized withdrawal design [10]. Importantly, almost half of participants were in remission, based on MADRS total score, at long-term follow-up. The remission rate of 46.1% at endpoint of the optimization/maintenance phase demonstrates that long-term treatment with esketamine provided sustained improvement of depressive symptoms in a substantial proportion of this treatment-resistant sample. Our findings extend those of Wajs et al. [14], who reported a 47.2% remission rate at 12 months. Persistent improvements in the mean clinician-assessed severity of illness (CGI) as well as patient-reported measures of depressive symptoms (PHQ-9), and functionality and associated disability (SDS) further support the long-term efficacy of esketamine for TRD. The relatively small proportions of participants who dropped out of the optimization/maintenance phase due to lack of efficacy (4.4%) or who discontinued due to worsening depression (0.6%) or suicide ideation (0.2%) suggest overall sustained efficacy during the study. These data compare favorably to the high proportions of participants in the STAR*D trial considered treatment resistant (based on nonresponse to at least 2 treatment regimens before responding to a third or fourth treatment regimen) who, following the 12- to 14-week acute treatment phase, manifested relapse rates of 65% and 71% during 1 year of continued treatment, with mean times to relapse of 3.1 and 3.3 months, for participants who had responded to the third or fourth treatment levels, respectively [5].

The generalizability of our findings is limited by the potential bias related to which participants chose to continue (or not continue) from the parent study into this study, the exclusion of participants with significant psychiatric or medical co-morbidities or substance dependence, and the relative lack of racial heterogeneity (86.8% white). Furthermore, sample size decreases at later study time points may have implications for representativeness and/or generalizability of findings. Notably, the data set includes current, interim data, which continues to be updated.

In summary, no new safety signal was identified and improvement of depression appeared to be sustained with long-term, intermittently-dosed esketamine in this study of participants with TRD.

## Supplementary information

Supplementary Material
