# Supplementary material for: Long-term safety and maintenance of response with esketamine nasal spray in participants with treatment-resistant depression: interim results of the SUSTAIN-3 study
Source: Neuropsychopharmacology. 2023 May 12;48(8):1225–33. doi: 10.1038/s41386-023-01577-5 (PMC10267177; doi:10.1038/s41386-023-01577-5)
Supplement: Supplementary file 1 — Supplementary Material [file 41386_2023_1577_MOESM1_ESM.pdf]

Contents of Online Supplementary Material

Figure S1. SUSTAIN-3 Study Design

Table S1. Characterization of the Study Population Entering SUSTAIN-3 from Different Phase 3 Studies

Table S2. Study Drug Dosing During the Optimization/Maintenance Phase of SUSTAIN-3

Table S3. Shift in Dosing Frequency of Esketamine

Table S4. Concomitant Oral Antidepressants Used in SUSTAIN-3

Table S5. Treatment-Emergent Serious Adverse Events During the Induction and Optimization/Maintenance Phases

Deaths

Modified Observer's Assessment of Alertness/Sedation (MOAA/S) Score

Summary of Participants Who Met Criteria for Markedly Elevated Blood Pressure

Figure S2. Incidence of Treatment-Emergent Increased Blood Pressure Events Over Time

Table S6. Mean Change from Study Baseline in Z-score Over Time Among Patients <65 Years Old (Induction and Optimization/Maintenance Phases of SUSTAIN-3)

Table S7. Mean Change from Study Baseline in Z-score Over Time Among Patients ≥65 Years Old (Induction and Optimization/Maintenance Phases of SUSTAIN-3)

Table S8. Most Severe Postbaseline Potentially Suicide-related Category vs. Baseline Based on Columbia-Suicide Severity Rating Scale (C-SSRS) Induction and Optimization/Maintenance Phases

Figure S3. Group Mean (± SE) Standardized (z-score) Change from Baseline for Psychomotor Function (Speed of Performance on the Detection Test) in SUSTAIN-3

Figure S4. Group Mean (± SE) Standardized (z-score) Change from Baseline for Attention Function (Speed of Performance on the Identification Test) in SUSTAIN-3

Figure S5. Mean (± SE) Patient Health Questionnaire (PHQ) Total Score (Observed Cases)

Figure S6. Remission Based on Sheehan Disability Scale (SDS) Score Over Time (Observed Cases)

Figure S1. SUSTAIN-3 Study Design

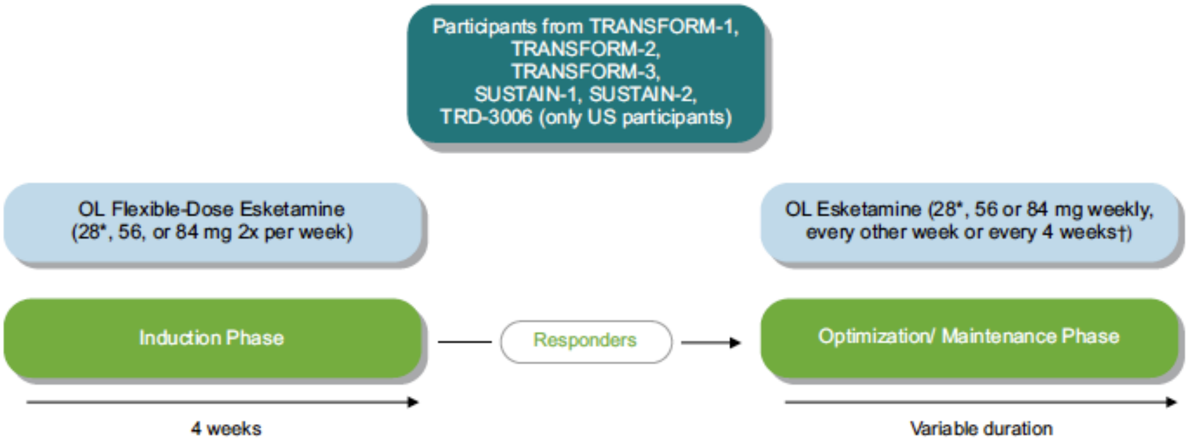

OL = open label; *28 mg dose only an option for patients ≥65 years; † Based on clinical global impression - severity (CGI-S) and tolerability
Note: Patients were eligible to enroll into the Induction Phase or the Optimization/Maintenance Phase based on their status at the end of the parent study (refer to Table S2).

Table S1. Characterization of the Study Population Entering SUSTAIN-3 from Different Phase 3 Studies

Prior Study ("Parent Study")	Inclusion Requirement	Point of Entry into SUSTAIN-3
TRANSFORM-1 (Fedgchin et al. 2019)	Participant completed induction phase and the 2-week follow-up phase visit.	Induction phase
	Participant completed the induction phase and was a responder and SUSTAIN-1 was terminated.	Optimization/maintenance phase
TRANSFORM-2 (Popova et al., 2019)	Participant completed induction phase and the 2-week follow-up phase visit.	Induction phase
	Participant completed the induction phase and was a responder and SUSTAIN-1 was terminated.	Optimization/maintenance phase
SUSTAIN-1 (Daly et al., 2019)	Participant relapsed in maintenance phase.	Induction phase
	Participant was in the induction phase at the time the study was terminated and, after completion of the induction phase, was determined to be a responder.	Optimization/maintenance phase
	Participant was in the optimization or maintenance phase at the time the study was terminated.	Optimization/maintenance phase
	At Week 16 of Optimization, the participant was not eligible to proceed to the maintenance phase and sponsor had approved participant's entry into SUSTAIN-3.	Induction phase or Optimization/maintenance phase
	Participant was in the induction phase and after completion of induction phase, did not meet criteria for response, and sponsor had approved participant's entry into SUSTAIN-3.	Induction phase or Optimization/maintenance phase
SUSTAIN-2 (Wajs et al., 2020)	Participant completed the optimization/maintenance phase.	Optimization/maintenance phase
	Participant was in the induction phase at the time the study was terminated and, after completion of the induction phase, was determined to be a responder.	Optimization/maintenance phase
	Participant was in the optimization/maintenance phase at the time the study was terminated.	Optimization/maintenance phase
	Participant was in the induction phase and did not meet criteria for response and sponsor had approved participant's entry into SUSTAIN-3.	Induction phase or Optimization/maintenance phase
TRANSFORM-3 (Ochs-Ross et al., 2020)	Participant was in the induction phase of TRANSFORM-3 at the time enrollment into SUSTAIN-2 was closed and, after completion of the induction phase, was determined to be a non-responder.	Induction phase
	Participant was in the induction phase of TRANSFORM-3 at the time enrollment into SUSTAIN-2 was closed and, after completion of the induction phase, was determined to be a responder.	Optimization/maintenance phase
TRD3006* (US study sites only)	Participant completed the induction phase and was a responder.	Optimization/maintenance phase
	Participant completed induction phase and did not meet the response criteria, and sponsor had approved participant's entry into the SUSTAIN-3 study.	Induction phase or Optimization/maintenance phase

Table S2. Study Drug Dosing During the Optimization/Maintenance Phase of SUSTAIN-3

For the first 4 weeks of the optimization/maintenance phase (week 1 to week 4):

- Participants from the induction phase of SUSTAIN-3, who entered the optimization/maintenance phase, continued on the same dose of esketamine from the induction phase and had a weekly treatment session frequency (i.e., reduced frequency from the twice-weekly frequency in the induction phase).
- Participants who were responders at the end of the induction phase of TRANSFORM-1, TRANSFORM-2, or Study TRD3006 (clinical trials.gov: NCT03434041; US sites only) who entered the optimization/maintenance phase had a weekly treatment session frequency (i.e., reduced frequency from the twice-weekly frequency in the induction phase). However, as the study drug in these 3 parent studies was blinded at the time of entry into SUSTAIN-3, participants who entered the optimization/maintenance phase were to start at 56 mg. The dose remained at 56 mg or was increased to 84 mg, as determined by the investigator based on efficacy and tolerability.
- With sponsor approval, participants from the SUSTAIN-1 and SUSTAIN-2 studies who entered directly from the induction phase, who did not meet the criteria for response in those studies entered the optimization/maintenance phase of SUSTAIN-3 and had a weekly treatment session frequency from week 1 to week 4 (i.e., reduced frequency from the twice-weekly frequency in the induction phase). In addition, a one-time dose change was allowed at study entry.
- With sponsor approval participants from Study TRD3006 (US sites only) who entered directly from induction phase and did not meet criteria for response in that study may have entered the optimization/maintenance phase. However, as the Study TRD3006 study drug was blinded at the time of entry into the current study, participants who entered the optimization/maintenance phase from Study TRD3006 started at 56 mg. The dose remained at 56 mg or was increased to 84 mg, as determined by the investigator based on efficacy and tolerability. Participants had a weekly treatment session frequency from week 1 to week 4 (i.e., reduced frequency from the twice-weekly frequency in the induction phase).
- Participants who entered the optimization/maintenance phase from the TRANSFORM-3 study also had a weekly treatment session frequency. However, as the TRANSFORM-3 study drug was blinded at the time of entry into SUSTAIN-3, the dose of esketamine was administered as outlined in Table 1.

Table 1. Optimization/Maintenance Phase Week 1 to 4: Dose Titration of Esketamine for Responder Participants Entering from TRANSFORM-3

Week	Dose	Dose Titration Guidance
Week 1	28 mg	
Week 2	28 or 56 mg	The dose may remain at 28 mg or be increased to 56 mg, as determined by the investigator based on efficacy and tolerability
Week 3 and 4	28, 56 or 84 mg	The dose may remain the same or be increased or reduced by 28 mg from the previous dosing session, as determined by the investigator based on efficacy and tolerability. For those who have had a prior down titration from a higher dose, a dose increase by 28 mg is allowed based on clinical judgment.

- Participants who entered the optimization/maintenance phase from SUSTAIN-1 (Direct Entry) or SUSTAIN-2 who were ongoing in the optimization, maintenance, or optimization/maintenance phase, respectively, had the option to have their current dosing frequency adjusted at the time of entry into SUSTAIN-3 and remained on the selected frequency from week 1 to week 4 (inclusive). A one-time dose change was permitted at study entry.

- Participants who entered the optimization/maintenance phase from SUSTAIN-1 (Transferred Entry) started at 56 mg. The dose remained at 56 mg or was increased to 84 mg, as determined by the investigator based on efficacy and tolerability. In addition, participants had the option to have their current dosing frequency adjusted at the time of entry into SUSTAIN-3 and remained on the selected frequency from week 1 to week 4 (inclusive).

After week 4 (i.e., starting at week 5), based on the investigator's clinical judgment, the dose of esketamine for all participants could be adjusted based upon efficacy and tolerability.

Starting at week 4, the frequency for subsequent treatment sessions was adjusted (if applicable) based on the algorithm outlined in Table 2 at fixed, 4-week intervals.

Table 2. Algorithm for Adjusting Treatment Session Frequency (if applicable) Starting Week 4

Current treatment session frequency	CGI-S score at current visit[a]	
	≤3	>3
Weekly	Change to every other week frequency	No change in frequency
Every other week	No change in frequency or change to every 4 weeks per clinical judgment	Change to weekly frequency
Every 4 weeks	No change in frequency	Change to weekly or every other week frequency per clinical judgment

[a] Note: Although the CGI-S is administered every 2 weeks from Week 4 through the end of the Optimization/Maintenance Phase, adjustment of the treatment session frequency is only permitted at the fixed, 4-week interval (based on CGI-S performed at that visit).

For example, if at week 4 a participant was currently at a weekly treatment session frequency and the CGI-S score at week 4 is a 2, the treatment session frequency was changed from weekly to every other week (i.e., the next treatment session for this participant will be at week 6).

Table S3. Shift in Dosing Frequency of Esketamine

a. First Year (N = 1083) – Dose Frequency Shift from Week 4 to Week 5-52

Baseline		Post (Week 5 – 52)		
		**Did Not Increase/Decrease**	**Increased Frequency at Least Once**	**Decreased Frequency at Least Once**
The first visit on or after Week 4	Every 4 Weeks	15/1083 (1.39%)	7/1083 (0.65%)	0/1083 (0%)
	Every Other Week	158/1083 (14.59%)	227/1083 (20.96%)	145/1083 (13.39%)
	Weekly	204/1083 (18.84%)	0/1083 (0%)	357/1083 (32.96%)

b. Second Year (N = 925) – Dose Frequency Shift from Week 52 to Week 53-104

Baseline		Post (Week 53-104)		
		**Did Not Increase/Decrease**	**Increased Frequency at Least Once**	**Decreased Frequency at Least Once**
The first visit on or after Week 52	Every 4 Weeks	86/925 (9.30%)	43/925 (4.65%)	0/925 (0%)
	Every Other Week	180/925 (19.46%)	156/925 (16.86%)	98/925 (10.59%)
	Weekly	231/925 (24.97%)	0/925 (0%)	157/925 (16.97%)

c. Third Year (N = 821) – Dose Frequency Shift from Week 104 to Week 105-

Baseline		Post (Week 105 -)		
		**Did Not Increase/Decrease**	**Increased Frequency at Least Once**	**Decreased Frequency at Least Once**
The first visit on or after Week 104	Every 4 Weeks	99/821 (12.06%)	40/821 (4.87%)	0/821 (0%)
	Every Other Week	160/821 (19.49%)	132/821 (16.08%)	64/821 (7.80%)
	Weekly	250/821 (30.45%)	0/821 (0%)	99/821 (12.06%)

Note "Did not Increase/Decrease" column refers to number of patients who did not switch their dosing frequency during the period (1st year, 2nd year, 3rd year).

Table S4. Concomitant Oral Antidepressants Used in SUSTAIN-3

**Induction Phase (N = 458)**	
Duloxetine	156 (34.1%)
Venlafaxine	86 (18.8%)
Escitalopram	79 (17.2%)
Sertraline	79 (17.2%)
Trazodone	27 (5.9%)
Bupropion	22 (4.8%)
Vortioxetine	20 (4.4%)
Mirtazapine	14 (3.1%)
Fluoxetine	11 (2.4%)
Paroxetine	7 (1.5%)
Citalopram	6 (1.3%)
Amitriptyline	4 (0.9%)
Nortriptyline	4 (0.9%)
Vilazodone	4 (0.9%)
Desvenlafaxine	3 (0.7%)
Fluvoxamine	3 (0.7%)
Levomilnacipran	3 (0.7%)
Clomipramine	2 (0.4%)
Imipramine	2 (0.4%)
Moclobemide	1 (0.2%)
Nefazodone	1 (0.2%)
Opipramol	1 (0.2%)
Tianeptine	1 (0.2%)
**Optimization/Maintenance Phase (N = 1110)**	
Duloxetine	399 (35.9%)
Venlafaxine	299 (26.9%)
Escitalopram	286 (25.8%)
Sertraline	202 (18.2%)
Bupropion	118 (10.6%)
Trazodone	87 (7.8%)
Vortioxetine	71 (6.4%)
Mirtazapine	63 (5.7%)
Fluoxetine	43 (3.9%)
Amitriptyline	29 (2.6%)
Desvenlafaxine	25 (2.3%)
Agomelatine	22 (2.0%)
Paroxetine	22 (2.0%)
Nortriptyline	16 (1.4%)
Vilazodone	16 (1.4%)
Citalopram	14 (1.3%)
Clomipramine	12 (1.1%)
Levomilnacipran	10 (0.9%)
Fluvoxamine	8 (0.7%)

Imipramine	5 (0.5%)
Tranylcypromine	4 (0.4%)
Desipramine	2 (0.2%)
Dosulepin	2 (0.2%)
Mianserin	2 (0.2%)
Nefazodone	2 (0.2%)
Reboxetine	2 (0.2%)
Doxepin	1 (0.1%)
Maprotiline hydrochloride	1 (0.1%)
Mianserin hydrochloride	1 (0.1%)
Moclobemide	1 (0.1%)
Opipramol	1 (0.1%)
Protriptyline	1 (0.1%)
Tianeptine	1 (0.1%)
Trimipramine	1 (0.1%)

Table S5. Treatment-Emergent Serious Adverse Events During the Induction and Optimization/Maintenance Phases

System Organ Class<br>Preferred Term	Esketamine Nasal Spray<br>(N = 1148)
Total no. participants with a serious adverse event	171 (14.9%)
Psychiatric disorders	49 (4.3%)
Depression*	17 (1.5%)
Suicide attempt	11 (1.0%)
Suicidal ideation	9 (0.8%)
Anxiety	5 (0.4%)
Major depression*	3 (0.3%)
Adjustment disorder	2 (0.2%)
Confusional state	2 (0.2%)
Affect lability	1 (0.1%)
Completed suicide	1 (0.1%)
Conversion disorder	1 (0.1%)
Depressed mood	1 (0.1%)
Depression suicidal	1 (0.1%)
Mania	1 (0.1%)
Persistent depressive disorder	1 (0.1%)
Infections and infestations	24 (2.1%)
Pneumonia	4 (0.3%)
COVID-19	3 (0.3%)
Cellulitis	3 (0.3%)
Cystitis	2 (0.2%)
Infection	2 (0.2%)
COVID-19 pneumonia	1 (0.1%)
Cellulitis staphylococcal	1 (0.1%)
Erysipelas	1 (0.1%)
Gastroenteritis	1 (0.1%)
Gastrointestinal infection	1 (0.1%)
Large intestine infection	1 (0.1%)
Otitis media chronic	1 (0.1%)
Pharyngitis streptococcal	1 (0.1%)
Postoperative wound infection	1 (0.1%)
Pyelonephritis	1 (0.1%)
Sepsis	1 (0.1%)
Staphylococcal infection	1 (0.1%)
Urinary tract infection	1 (0.1%)
Injury, poisoning and procedural complications	23 (2.0%)

System Organ Class<br>Preferred Term	Esketamine Nasal Spray<br>(N = 1148)
Lower limb fracture	3 (0.3%)
Ankle fracture	2 (0.2%)
Fall	2 (0.2%)
Wrist fracture	2 (0.2%)
Alcohol poisoning	1 (0.1%)
Animal bite	1 (0.1%)
Exposure during pregnancy	1 (0.1%)
Face injury	1 (0.1%)
Femur fracture	1 (0.1%)
Incisional hernia	1 (0.1%)
Intentional overdose	1 (0.1%)
Ligament rupture	1 (0.1%)
Meniscus injury	1 (0.1%)
Multiple injuries	1 (0.1%)
Muscle rupture	1 (0.1%)
Overdose	1 (0.1%)
Pelvic fracture	1 (0.1%)
Road traffic accident	1 (0.1%)
Skin laceration	1 (0.1%)
Tibia fracture	1 (0.1%)
Traumatic intracranial haemorrhage	1 (0.1%)
Gastrointestinal disorders	14 (1.2%)
Ileus	2 (0.2%)
Pancreatitis acute	2 (0.2%)
Umbilical hernia	2 (0.2%)
Colitis	1 (0.1%)
Constipation	1 (0.1%)
Diarrhoea	1 (0.1%)
Haemorrhoids	1 (0.1%)
Hiatus hernia	1 (0.1%)
Inguinal hernia	1 (0.1%)
Large intestine polyp	1 (0.1%)
Lower gastrointestinal haemorrhage	1 (0.1%)
Nervous system disorders	14 (1.2%)
Headache	3 (0.3%)
Carotid artery aneurysm	2 (0.2%)
Cerebrovascular accident	2 (0.2%)
Akathisia	1 (0.1%)
Dysarthria	1 (0.1%)
Encephalopathy	1 (0.1%)
Facial paralysis	1 (0.1%)
Hemiparesis	1 (0.1%)

**System Organ Class**<br>**Preferred Term**	**Esketamine Nasal Spray**<br>**(N = 1148)**
Hemiplegia	1 (0.1%)
Intracranial aneurysm	1 (0.1%)
Ischaemic stroke	1 (0.1%)
Loss of consciousness	1 (0.1%)
Metabolic encephalopathy	1 (0.1%)
Occipital neuralgia	1 (0.1%)
Seizure	1 (0.1%)
Transient ischaemic attack	1 (0.1%)
Renal and urinary disorders	13 (1.1%)
Nephrolithiasis	5 (0.4%)
Stress urinary incontinence	2 (0.2%)
Acute kidney injury	1 (0.1%)
Bladder outlet obstruction	1 (0.1%)
Chronic kidney disease	1 (0.1%)
Renal artery stenosis	1 (0.1%)
Renal failure	1 (0.1%)
Renal mass	1 (0.1%)
Urethral stenosis	1 (0.1%)
Urge incontinence	1 (0.1%)
Urinary bladder polyp	1 (0.1%)
Cardiac disorders	12 (1.0%)
Atrial fibrillation	4 (0.3%)
Myocardial infarction	3 (0.3%)
Acute myocardial infarction	1 (0.1%)
Arrhythmia	1 (0.1%)
Bradycardia	1 (0.1%)
Coronary artery disease	1 (0.1%)
Pericardial effusion	1 (0.1%)
Respiratory, thoracic and mediastinal disorders	12 (1.0%)
Asthma	2 (0.2%)
Nasal polyps	2 (0.2%)
Bronchitis chronic	1 (0.1%)
Dyspnoea	1 (0.1%)
Dyspnoea exertional	1 (0.1%)
Lung disorder	1 (0.1%)
Nasal septum deviation	1 (0.1%)
Pneumothorax spontaneous	1 (0.1%)
Pulmonary embolism	1 (0.1%)
Pulmonary oedema	1 (0.1%)

System Organ Class<br>Preferred Term	Esketamine Nasal Spray<br>(N = 1148)
Neoplasms benign, malignant and unspecified (incl cysts and polyps)	11 (1.0%)
Breast cancer	2 (0.2%)
Prostate cancer	2 (0.2%)
Anal cancer	1 (0.1%)
Breast neoplasm	1 (0.1%)
Invasive breast carcinoma	1 (0.1%)
Lung adenocarcinoma	1 (0.1%)
Lymphoma	1 (0.1%)
Oesophageal squamous cell carcinoma	1 (0.1%)
Squamous cell carcinoma	1 (0.1%)
Musculoskeletal and connective tissue disorders	10 (0.9%)
Back pain	3 (0.3%)
Intervertebral disc degeneration	2 (0.2%)
Intervertebral disc protrusion	2 (0.2%)
Osteoarthritis	2 (0.2%)
Arthritis	1 (0.1%)
Cervical spinal stenosis	1 (0.1%)
Intervertebral disc disorder	1 (0.1%)
Rotator cuff syndrome	1 (0.1%)
Spondylolisthesis	1 (0.1%)
Hepatobiliary disorders	9 (0.8%)
Cholelithiasis	7 (0.6%)
Bile duct stone	1 (0.1%)
Biliary obstruction	1 (0.1%)
Cholecystitis	1 (0.1%)
Reproductive system and breast disorders	6 (0.5%)
Breast hyperplasia	1 (0.1%)
Cervical dysplasia	1 (0.1%)
Endometriosis	1 (0.1%)
Epididymal cyst	1 (0.1%)
Menorrhagia	1 (0.1%)
Vaginal prolapse	1 (0.1%)
Metabolism and nutrition disorders	5 (0.4%)
Hyperglycaemia	2 (0.2%)
Diabetic ketoacidosis	1 (0.1%)
Hypercalcaemia	1 (0.1%)
Obesity	1 (0.1%)
Type 2 diabetes mellitus	1 (0.1%)

System Organ Class<br>Preferred Term	Esketamine Nasal Spray<br>(N = 1148)
Ear and labyrinth disorders	3 (0.3%)
Vertigo	2 (0.2%)
Vertigo positional	1 (0.1%)
General disorders and administration site conditions	3 (0.3%)
Asthenia	1 (0.1%)
Fatigue	1 (0.1%)
Pyrexia	1 (0.1%)
Vascular disorders	3 (0.3%)
Circulatory collapse	1 (0.1%)
Hypertensive emergency	1 (0.1%)
Orthostatic hypotension	1 (0.1%)
Investigations	2 (0.2%)
Myocardial necrosis marker increased	1 (0.1%)
SARS-CoV-2 test	1 (0.1%)
Immune system disorders	1 (0.1%)
Anaphylactic reaction	1 (0.1%)
Product issues	1 (0.1%)
Device breakage	1 (0.1%)
Surgical and medical procedures	1 (0.1%)
Female sterilisation	1 (0.1%)

*Treatment-emergent adverse events are defined as events with onset during treatment or that were a consequence of a pre-existing condition that had worsened since baseline. All adverse events are reported as preferred terms, coded using the Medical Dictionary for Regulatory Activities (MedDRA) version 23.1. Thus, verbatim reporting of "worsening of depression", "relapse of depression", or "hospitalization due to depression" as adverse events were coded and are reported as depression, and "worsening of major depression" or "relapse of major depression", etc. as major depression.

Note: Incidence is based on the number of participants experiencing at least one adverse event, not the number of events.

Deaths

There were 5 (0.4%) deaths, none considered by the investigator as related to esketamine. The deaths included: one suicide, multiple injuries secondary to a bike accident (59-year-old male, death on day 364, 6 days after last dose of esketamine), myocardial infarction (73-year-old female, death on day 321, 6 days after last dose of esketamine), and two due to COVID-19 (66-year-old male, death on day 927, 13 days after last dose of esketamine; and, 60-year-old male, death on day 1052, 24 days after last dose of esketamine).

Modified Observer's Assessment of Alertness/Sedation (MOAA/S) Score

The MOAA/S, scored from 0 (no response to painful stimuli) to 5 (readily responds to name spoken in normal tone) was performed every 15 minutes from predose to 1 hour postdose on each dosing day.

Clinically-relevant sedation, defined by MOAA/S score ≤ 3 (moderate sedation: responds after name called loudly or repeatedly), occurred in 6.1% (28/458) of patients in the induction phase and 6.9% (77/1110) of patients in the optimization/maintenance phase.

The MOAA/S score decreased within 15 minutes of dosing, plateaued at 30 to 45 minutes, and returned to near predose levels by 1 hour during both the induction and optimization/maintenance phases.

Summary of Participants Who Met Criteria for Markedly Elevated Blood Pressure

- A minority (6.3%, 72/1148) of participants met the study criteria for markedly elevated blood pressure (i.e., systolic blood pressure ≥180 mmHg or diastolic blood pressure ≥110 mmHg) at any time during the combined induction and optimization/maintenance phases, with rate higher among those with versus without history of hypertension (systolic blood pressure ≥180 mmHg: 7.0% vs.1.5%; diastolic blood pressure ≥110 mmHg: 9.2% vs. 3.2%, respectively).
- All occurrences of elevated blood pressure on a dosing day were transient and resolved the same day. Seven (0.6%) participants had elevated blood pressure that did not occur on a dosing day but resolved the same day.
- Percent of Treatment Episodes with Abnormal Blood Pressure:
  - **All Participants**: The number of occurrences as a percent of treatment episodes for post-dose systolic blood pressure ≥180 mmHg at any time was 0.11% in the induction phase and 0.04% in the optimization/maintenance phase, and for post-dose diastolic blood pressure ≥110 mmHg at any time was 0.65% and 0.06% for the respective phases.
  - **Participants with Hypertension at Baseline**: The number of occurrences as a percent of treatment episodes for post-dose systolic blood pressure ≥180 mmHg at any time was 0.43% in the induction phase and 0.08% in the optimization/maintenance phase, and for post-dose diastolic blood pressure ≥110 mmHg at any time was 1.59% and 0.11% for the respective phases.

Figure S2. Incidence of Treatment-Emergent Increased Blood Pressure Events Over Time

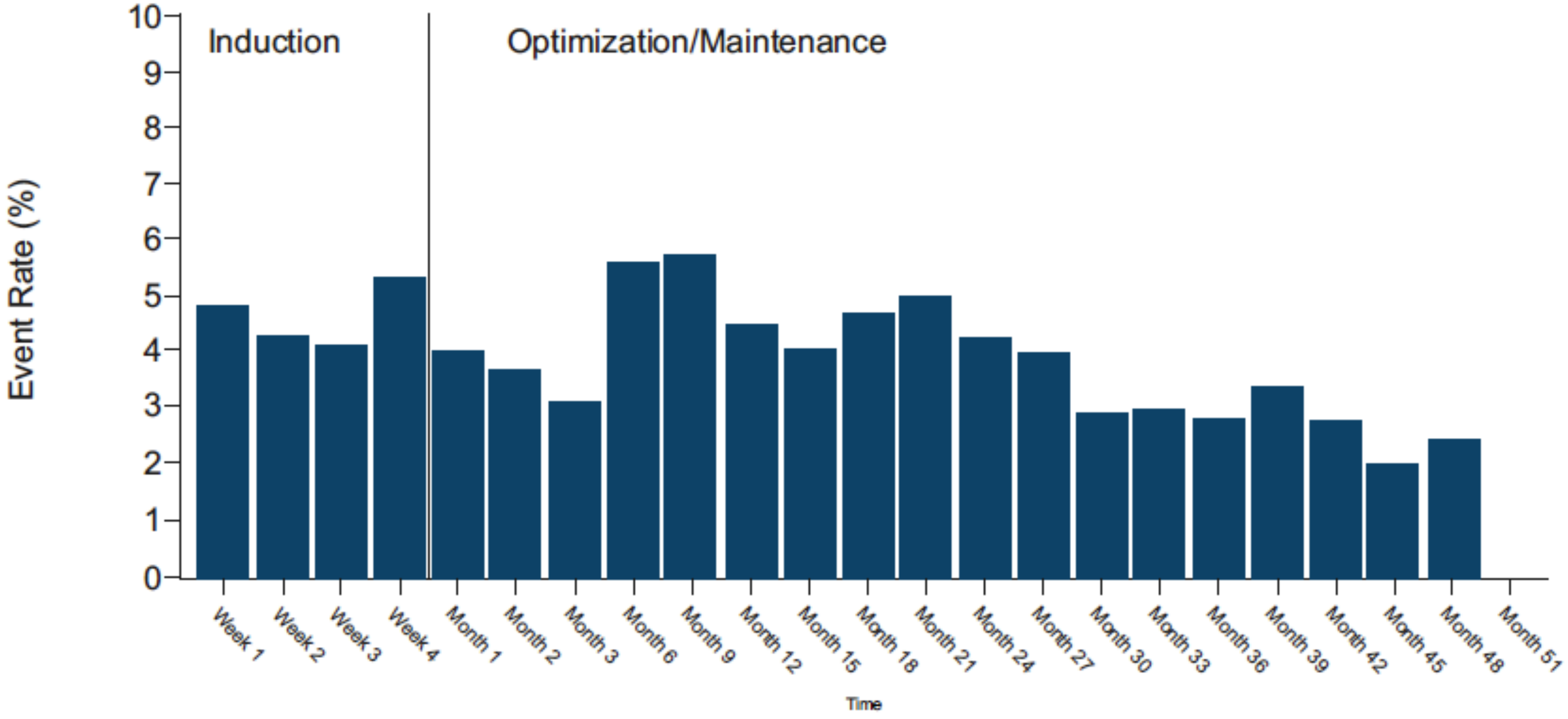

No. of Participants with AEs	22	19	18	23	44	39	32	58	57	43	37	42	44	36	33	23	23	21	23	14	5	2	0
No. of Participants Dosed	458	447	443	434	1110	1077	1054	1042	997	968	932	902	883	854	835	809	784	753	693	517	260	83	25

Notes: Adverse events rate = number of participants dosed and with treatment-emergent adverse event(s) in interval / number of participants dosed within interval.
Study week (or month) is defined using the phase study day by every 7 days (or 28 days).
The intervals with the number of participants <10 are not presented.

Table S6. Mean Change from Study Baseline in Z-score Over Time Among Participants < 65 Years Old (Induction and Optimization/Maintenance Phases of SUSTAIN-3)

							Baseline	Change from Baseline						
**Task**	**N**	**Mean**	**SD**	**Median**	**Min**	**Max**	**Mean (SD)**	**N**	**Mean***	**SE**	**SD**	**Median***	**Min**	**Max**
Detection														
Baseline (IND)	1003	-0.765	1.3880	-0.586	-5.94	2.37								
Day 28 (IND)	408	-0.614	1.3518	-0.350	-5.96	2.05	-0.826 (1.4840)	408	0.212	0.0498	1.0052	0.133	-3.23	6.28
Endpoint (IND)	408	-0.616	1.3522	-0.350	-5.96	2.05	-0.826 (1.4840)	408	0.210	0.0498	1.0058	0.128	-3.23	6.28
Week 3 (OP/MA)	926	-0.708	1.3259	-0.569	-6.67	1.92	-0.788 (1.3963)	917	0.075	0.0342	1.0347	0.042	-5.70	5.16
Week 16 (OP/MA)	871	-0.738	1.3545	-0.587	-6.56	1.85	-0.804 (1.3963)	865	0.065	0.0374	1.1000	0.055	-4.85	5.21
Week 28 (OP/MA)	858	-0.746	1.3716	-0.648	-5.89	2.03	-0.783 (1.3752)	852	0.034	0.0398	1.1613	0.004	-6.07	5.11
Week 40 (OP/MA)	831	-0.729	1.3008	-0.587	-5.85	2.09	-0.786 (1.3894)	824	0.057	0.0402	1.1544	0.015	-4.21	5.19
Week 52 (OP/MA)	806	-0.772	1.3433	-0.622	-6.74	2.02	-0.788 (1.3795)	800	0.013	0.0422	1.1942	0.010	-7.23	5.77
Week 64 (OP/MA)	769	-0.789	1.3282	-0.643	-6.16	1.94	-0.790 (1.3749)	762	-0.003	0.0425	1.1721	-0.010	-5.11	4.75
Week 76 (OP/MA)	758	-0.736	1.2816	-0.611	-5.68	2.17	-0.791 (1.3857)	752	0.052	0.0445	1.2201	0.035	-4.75	5.04
Week 88 (OP/MA)	727	-0.788	1.3684	-0.684	-6.56	1.85	-0.788 (1.4011)	722	-0.005	0.0452	1.2148	-0.057	-5.04	5.23
Week 100 (OP/MA)	716	-0.763	1.3773	-0.663	-6.35	2.07	-0.800 (1.3899)	713	0.036	0.0486	1.2970	0.002	-5.09	5.18
Week 112 (OP/MA)	672	-0.787	1.3340	-0.776	-6.37	1.96	-0.783 (1.3960)	670	-0.005	0.0488	1.2622	-0.036	-5.02	4.78
Week 124 (OP/MA)	662	-0.843	1.3485	-0.797	-6.88	2.02	-0.806 (1.4121)	662	-0.037	0.0499	1.2830	-0.073	-4.84	5.34
Week 136 (OP/MA)	617	-0.741	1.2649	-0.637	-5.91	2.21	-0.799 (1.4056)	617	0.058	0.0493	1.2248	0.030	-6.24	5.18
Week 148 (OP/MA)	509	-0.805	1.2827	-0.674	-6.03	2.09	-0.785 (1.3325)	509	-0.019	0.0588	1.3267	-0.070	-6.36	4.43
Week 160 (OP/MA)	386	-0.794	1.3154	-0.704	-6.77	2.08	-0.722 (1.2998)	386	-0.072	0.0644	1.2645	-0.061	-7.39	4.17
Week 172 (OP/MA)	156	-0.505	1.2126	-0.445	-6.39	1.97	-0.574 (1.2765)	156	0.069	0.0908	1.1339	0.002	-3.13	4.35
Week 184 (OP/MA)	52	-0.217	1.1309	-0.100	-2.85	1.99	-0.197 (1.1868)	52	-0.020	0.1333	0.9615	0.057	-1.52	2.60
Week 196 (OP/MA)	14	0.076	1.0847	0.088	-2.21	1.45	-0.358 (1.2965)	14	0.434	0.3999	1.4962	0.194	-1.95	3.94
Week 208 (OP/MA)	1	0.485	-	0.485	0.49	0.49	0.900 (-)	1	-0.415	-	-	-0.415	-0.42	-0.42
Week 220 (OP/MA)	1	-0.149	-	-0.149	-0.15	-0.15	0.643 (-)	1	-0.792	-	-	-0.792	-0.79	-0.79
Endpoint (OP/MA)	975	-0.818	1.3510	-0.725	-6.77	1.99	-0.768 (1.3881)	966	-0.054	0.0403	1.2539	-0.071	-7.39	5.18
Identification														
Baseline (IND)	1003	-0.649	1.4087	-0.535	-6.19	3.00								
Day 28 (IND)	410	-0.541	1.4199	-0.311	-5.95	2.50	-0.640 (1.5433)	410	0.100	0.0513	1.0395	0.071	-2.75	5.59
Endpoint (IND)	410	-0.545	1.4269	-0.311	-5.95	2.50	-0.640 (1.5433)	410	0.095	0.0518	1.0493	0.071	-3.19	5.59
Week 3 (OP/MA)	930	-0.672	1.3740	-0.555	-6.21	2.37	-0.666 (1.4077)	921	-0.017	0.0351	1.0643	-0.039	-4.98	6.19
Week 16 (OP/MA)	880	-0.706	1.3748	-0.596	-6.31	2.64	-0.689 (1.4127)	874	-0.023	0.0363	1.0723	-0.035	-4.72	6.48
Week 28 (OP/MA)	866	-0.737	1.4715	-0.568	-6.75	2.24	-0.658 (1.3885)	860	-0.087	0.0406	1.1906	-0.066	-7.41	6.73
Week 40 (OP/MA)	838	-0.723	1.3626	-0.588	-5.78	2.70	-0.680 (1.4016)	831	-0.051	0.0407	1.1739	-0.100	-4.70	6.57
Week 52 (OP/MA)	810	-0.748	1.4445	-0.532	-6.29	3.26	-0.672 (1.3913)	804	-0.084	0.0443	1.2559	-0.068	-4.94	6.86
Week 64 (OP/MA)	774	-0.765	1.3715	-0.627	-6.98	2.36	-0.686 (1.3773)	767	-0.089	0.0449	1.2441	-0.134	-6.86	5.70
Week 76 (OP/MA)	759	-0.760	1.4363	-0.599	-6.68	2.89	-0.679 (1.3900)	753	-0.082	0.0449	1.2320	-0.098	-5.85	6.04
Week 88 (OP/MA)	730	-0.772	1.4587	-0.608	-6.35	2.60	-0.674 (1.4164)	725	-0.101	0.0445	1.1979	-0.133	-4.92	5.98
Week 100 (OP/MA)	719	-0.832	1.4406	-0.695	-6.06	2.24	-0.692 (1.3928)	716	-0.142	0.0471	1.2615	-0.178	-5.21	5.88

							Baseline	Change from Baseline						
**Task**	**N**	**Mean**	**SD**	**Median**	**Min**	**Max**	**Mean (SD)**	**N**	**Mean***	**SE**	**SD**	**Median***	**Min**	**Max**
Week 112 (OP/MA)	676	-0.828	1.4268	-0.724	-6.57	2.75	-0.683 (1.4045)	674	-0.147	0.0507	1.3173	-0.173	-5.37	5.83
Week 124 (OP/MA)	666	-0.818	1.4535	-0.670	-6.65	2.46	-0.714 (1.4378)	666	-0.104	0.0500	1.2894	-0.159	-5.40	6.50
Week 136 (OP/MA)	622	-0.793	1.3673	-0.672	-6.87	2.57	-0.698 (1.4003)	622	-0.095	0.0517	1.2891	-0.139	-6.19	5.78
Week 148 (OP/MA)	511	-0.795	1.3209	-0.702	-4.97	2.59	-0.679 (1.3467)	511	-0.116	0.0577	1.3038	-0.163	-3.97	6.12
Week 160 (OP/MA)	386	-0.828	1.3620	-0.701	-6.02	2.52	-0.616 (1.2830)	386	-0.212	0.0631	1.2402	-0.186	-5.24	3.93
Week 172 (OP/MA)	156	-0.650	1.3678	-0.495	-6.35	2.73	-0.514 (1.2974)	156	-0.136	0.0927	1.1573	-0.200	-3.61	3.48
Week 184 (OP/MA)	52	-0.262	1.1608	-0.073	-3.64	2.42	-0.066 (1.2561)	52	-0.196	0.1294	0.9334	-0.269	-2.10	3.26
Week 196 (OP/MA)	17	0.114	1.1316	0.638	-2.84	1.34	0.227 (1.2294)	17	-0.113	0.2251	0.9280	0.144	-2.65	1.15
Week 208 (OP/MA)	1	-0.252	-	-0.252	-0.25	-0.25	1.696 (-)	1	-1.948	-	-	-1.948	-1.95	-1.95
Week 220 (OP/MA)	1	0.760	-	0.760	0.76	0.76	0.700 (-)	1	0.060	-	-	0.060	0.06	0.06
Endpoint (OP/MA)	974	-0.864	1.3999	-0.747	-6.98	2.42	-0.655 (1.4083)	965	-0.220	0.0423	1.3152	-0.232	-7.41	6.12
One Card Learning														
Baseline (IND)	1003	-0.032	1.2728	-0.123	-4.42	3.99								
Day 28 (IND)	417	0.025	1.2351	0.117	-4.68	3.05	-0.122 (1.1721)	417	0.147	0.0525	1.0718	0.158	-5.25	4.69
Endpoint (IND)	417	0.026	1.2351	0.117	-4.68	3.05	-0.122 (1.1721)	417	0.148	0.0525	1.0722	0.158	-5.25	4.69
Week 3 (OP/MA)	934	0.068	1.2900	0.073	-4.58	3.99	-0.029 (1.2658)	925	0.102	0.0380	1.1548	0.037	-5.94	5.36
Week 16 (OP/MA)	886	0.060	1.3345	0.073	-5.82	3.99	-0.043 (1.2801)	880	0.106	0.0380	1.1261	0.111	-6.76	4.95
Week 28 (OP/MA)	868	0.124	1.3035	0.120	-4.84	3.74	0.001 (1.2627)	862	0.124	0.0378	1.1098	0.044	-5.35	4.42
Week 40 (OP/MA)	841	0.186	1.3387	0.151	-5.82	4.01	-0.002 (1.2599)	834	0.185	0.0405	1.1684	0.164	-7.05	5.33
Week 52 (OP/MA)	814	0.215	1.3369	0.279	-4.20	3.99	-0.013 (1.2605)	808	0.226	0.0404	1.1497	0.214	-4.31	4.57
Week 64 (OP/MA)	776	0.199	1.4388	0.247	-4.96	4.01	-0.014 (1.2657)	769	0.214	0.0449	1.2457	0.239	-6.17	5.25
Week 76 (OP/MA)	762	0.226	1.3430	0.247	-5.16	4.67	-0.017 (1.2707)	756	0.249	0.0448	1.2331	0.265	-7.06	4.92
Week 88 (OP/MA)	729	0.259	1.4248	0.310	-5.20	4.67	-0.005 (1.2735)	724	0.263	0.0460	1.2372	0.273	-5.19	4.65
Week 100 (OP/MA)	719	0.234	1.4297	0.230	-7.03	5.01	-0.020 (1.2747)	716	0.259	0.0499	1.3344	0.264	-9.11	5.31
Week 112 (OP/MA)	676	0.315	1.4688	0.280	-3.64	5.01	-0.003 (1.2879)	674	0.320	0.0498	1.2924	0.343	-4.34	4.60
Week 124 (OP/MA)	670	0.342	1.4436	0.380	-5.09	4.67	0.006 (1.2766)	670	0.335	0.0485	1.2557	0.299	-6.02	4.87
Week 136 (OP/MA)	627	0.281	1.4556	0.369	-4.91	4.67	-0.004 (1.2762)	627	0.286	0.0504	1.2628	0.280	-4.93	4.26
Week 148 (OP/MA)	513	0.346	1.4793	0.431	-6.54	4.67	-0.019 (1.2957)	513	0.364	0.0584	1.3238	0.386	-6.92	4.48
Week 160 (OP/MA)	387	0.408	1.4649	0.431	-6.54	4.67	0.066 (1.2679)	387	0.343	0.0688	1.3540	0.412	-8.90	4.31
Week 172 (OP/MA)	157	0.479	1.4889	0.514	-5.99	3.56	0.157 (1.3038)	157	0.322	0.1088	1.3631	0.418	-8.28	3.34
Week 184 (OP/MA)	51	0.778	1.4294	0.936	-1.70	3.35	0.300 (1.2947)	51	0.478	0.1606	1.1472	0.693	-2.92	2.35
Week 196 (OP/MA)	16	1.196	0.9078	1.128	-0.87	2.50	0.750 (1.0388)	16	0.447	0.2465	0.9860	0.252	-1.11	2.65
Week 208 (OP/MA)	2	1.995	1.4906	1.995	0.94	3.05	0.436 (0.3466)	2	1.560	0.8089	1.1440	1.560	0.75	2.37
Week 220 (OP/MA)	1	0.583	-	0.583	0.58	0.58	0.191 (-)	1	0.392	-	-	0.392	0.39	0.39
Endpoint (OP/MA)	975	0.251	1.4317	0.343	-6.54	4.67	-0.025 (1.2681)	966	0.277	0.0431	1.3386	0.272	-8.90	5.25
One-Back														
Baseline (IND)	1003	-0.503	1.2913	-0.465	-5.56	2.94								
Day 28 (IND)	416	-0.496	1.3607	-0.424	-5.69	3.25	-0.586 (1.3536)	416	0.090	0.0441	0.8985	0.057	-3.76	4.01
Endpoint (IND)	416	-0.498	1.3610	-0.424	-5.69	3.25	-0.586 (1.3536)	416	0.089	0.0441	0.8996	0.057	-3.76	4.01
Week 3 (OP/MA)	936	-0.409	1.3314	-0.281	-5.48	3.43	-0.508 (1.2932)	927	0.093	0.0307	0.9336	0.088	-4.89	4.66

							Baseline	Change from Baseline						
**Task**	**N**	**Mean**	**SD**	**Median**	**Min**	**Max**	**Mean (SD)**	**N**	**Mean***	**SE**	**SD**	**Median***	**Min**	**Max**
Week 16 (OP/MA)	886	-0.445	1.2790	-0.400	-5.22	3.91	-0.528 (1.2942)	880	0.076	0.0313	0.9270	0.085	-4.06	5.33
Week 28 (OP/MA)	868	-0.442	1.2791	-0.369	-5.22	3.82	-0.503 (1.2632)	862	0.056	0.0335	0.9844	0.016	-5.75	7.78
Week 40 (OP/MA)	842	-0.428	1.2573	-0.398	-4.54	3.15	-0.511 (1.2561)	835	0.081	0.0349	1.0082	0.049	-3.92	4.80
Week 52 (OP/MA)	814	-0.424	1.2895	-0.364	-5.41	3.19	-0.508 (1.2545)	808	0.077	0.0368	1.0470	0.077	-4.83	6.86
Week 64 (OP/MA)	776	-0.436	1.2453	-0.300	-4.79	3.30	-0.528 (1.2339)	769	0.084	0.0370	1.0258	0.082	-3.75	4.48
Week 76 (OP/MA)	764	-0.388	1.2523	-0.210	-5.60	2.59	-0.522 (1.2453)	758	0.132	0.0359	0.9898	0.147	-4.19	4.35
Week 88 (OP/MA)	731	-0.384	1.2699	-0.324	-5.24	2.97	-0.512 (1.2542)	726	0.127	0.0387	1.0416	0.124	-5.24	4.55
Week 100 (OP/MA)	720	-0.336	1.2235	-0.302	-4.36	2.66	-0.530 (1.2306)	717	0.194	0.0385	1.0313	0.173	-3.55	4.37
Week 112 (OP/MA)	677	-0.352	1.2612	-0.272	-4.98	3.27	-0.520 (1.2432)	675	0.166	0.0412	1.0709	0.154	-4.07	4.66
Week 124 (OP/MA)	670	-0.326	1.2627	-0.280	-4.72	3.32	-0.542 (1.2651)	670	0.215	0.0433	1.1213	0.253	-5.27	5.01
Week 136 (OP/MA)	629	-0.310	1.2718	-0.269	-5.15	3.06	-0.524 (1.2250)	629	0.214	0.0429	1.0762	0.177	-3.95	6.39
Week 148 (OP/MA)	514	-0.267	1.1832	-0.216	-4.60	2.65	-0.528 (1.1912)	514	0.261	0.0479	1.0859	0.285	-4.51	6.35
Week 160 (OP/MA)	387	-0.235	1.2203	-0.227	-5.21	3.02	-0.513 (1.2119)	387	0.279	0.0522	1.0276	0.254	-3.84	4.19
Week 172 (OP/MA)	157	-0.053	1.3586	0.042	-6.13	2.93	-0.286 (1.2846)	157	0.234	0.0898	1.1252	0.203	-3.47	3.98
Week 184 (OP/MA)	52	0.397	1.1296	0.389	-2.45	2.37	0.292 (1.3113)	52	0.105	0.1346	0.9707	0.090	-1.70	4.14
Week 196 (OP/MA)	17	0.569	1.5485	0.619	-3.16	2.57	0.473 (1.4257)	17	0.096	0.2203	0.9085	0.228	-2.24	1.69
Week 208 (OP/MA)	2	0.274	0.2393	0.274	0.11	0.44	-0.023 (0.8957)	2	0.298	0.8026	1.1351	0.298	-0.51	1.10
Week 220 (OP/MA)	1	0.507	-	0.507	0.51	0.51	-0.657 (-)	1	1.163	-	-	1.163	1.16	1.16
Endpoint (OP/MA)	975	-0.339	1.3128	-0.302	-6.13	3.19	-0.507 (1.2966)	966	0.164	0.0348	1.0826	0.176	-3.57	6.35
Groton Maze Learning Test														
Baseline (IND)	996	0.140	1.0725	0.316	-12.83	2.05								
Day 28 (IND)	403	0.193	1.1218	0.417	-10.69	1.67	0.118 (1.1604)	403	0.075	0.0396	0.7943	0.035	-3.78	5.07
Endpoint (IND)	403	0.194	1.1181	0.417	-10.69	1.67	0.118 (1.1604)	403	0.076	0.0395	0.7935	0.035	-3.78	5.07
Week 3 (OP/MA)	920	0.209	1.1066	0.417	-13.31	2.13	0.172 (0.9722)	908	0.045	0.0267	0.8032	0.087	-7.11	3.86
Week 16 (OP/MA)	873	0.208	1.0929	0.417	-10.24	1.92	0.157 (1.0818)	863	0.054	0.0294	0.8645	0.069	-11.67	4.73
Week 28 (OP/MA)	854	0.278	1.0562	0.462	-11.09	2.11	0.173 (1.0664)	844	0.111	0.0281	0.8154	0.117	-8.96	3.97
Week 40 (OP/MA)	828	0.245	1.0919	0.443	-11.85	2.05	0.170 (1.0500)	817	0.081	0.0300	0.8578	0.089	-5.71	4.72
Week 52 (OP/MA)	800	0.303	1.0304	0.462	-10.55	2.18	0.163 (1.0866)	789	0.148	0.0284	0.7989	0.134	-2.48	5.15
Week 64 (OP/MA)	766	0.331	0.9154	0.514	-5.94	2.22	0.171 (1.0798)	755	0.165	0.0320	0.8804	0.130	-2.95	9.63
Week 76 (OP/MA)	756	0.306	0.9828	0.509	-6.79	1.96	0.169 (1.0823)	745	0.145	0.0338	0.9233	0.134	-3.94	12.09
Week 88 (OP/MA)	723	0.370	0.9046	0.546	-4.38	1.98	0.168 (1.1026)	715	0.207	0.0335	0.8946	0.173	-2.87	11.46
Week 100 (OP/MA)	712	0.363	1.0386	0.551	-8.77	2.18	0.175 (1.0901)	705	0.193	0.0298	0.7903	0.173	-3.32	4.07
Week 112 (OP/MA)	676	0.310	0.9925	0.511	-6.72	2.05	0.156 (1.1133)	671	0.160	0.0386	0.9999	0.139	-5.53	12.58
Week 124 (OP/MA)	660	0.361	1.0324	0.546	-9.98	2.13	0.177 (1.0947)	656	0.189	0.0403	1.0326	0.139	-4.63	12.80
Week 136 (OP/MA)	621	0.386	0.9502	0.580	-6.20	2.09	0.151 (1.1297)	618	0.236	0.0365	0.9082	0.178	-3.26	10.75
Week 148 (OP/MA)	511	0.354	0.9956	0.551	-5.43	2.00	0.152 (1.1167)	508	0.208	0.0452	1.0190	0.178	-4.06	12.44
Week 160 (OP/MA)	384	0.431	0.9306	0.580	-3.64	2.02	0.179 (1.1325)	382	0.252	0.0522	1.0206	0.220	-3.77	11.91
Week 172 (OP/MA)	157	0.549	0.7647	0.644	-2.06	2.05	0.360 (0.8807)	156	0.187	0.0502	0.6271	0.196	-1.61	3.20
Week 184 (OP/MA)	52	0.530	0.8841	0.577	-3.03	1.84	0.388 (0.7705)	52	0.142	0.0920	0.6638	0.201	-2.12	1.50
Week 196 (OP/MA)	17	0.748	0.6506	0.774	-0.56	1.83	0.637 (0.6702)	17	0.111	0.1189	0.4903	0.045	-1.16	0.83

Task	N	Mean	SD	Median	Min	Max	Baseline Mean (SD)	Change from Baseline N	Mean*	SE	SD	Median*	Min	Max
Week 208 (OP/MA)	2	1.042	0.4415	1.042	0.73	1.35	0.997 (0.6308)	2	0.045	0.1338	0.1892	0.045	-0.09	0.18
Week 220 (OP/MA)	1	1.399	-	1.399	1.40	1.40	1.443 (-)	1	-0.045	-	-	-0.045	-0.04	-0.04
Endpoint (OP/MA)	977	0.309	1.0779	0.546	-10.24	2.11	0.150 (1.0694)	961	0.168	0.0334	1.0342	0.178	-11.67	11.91
HVLT-R, Word recall														
Baseline (IND)	1007	-0.085	1.2220	0.109	-4.38	2.10								
Day 28 (IND)	430	-0.091	1.2269	-0.009	-4.15	2.09	-0.078 (1.2137)	430	-0.013	0.0462	0.9584	0.000	-3.55	3.20
Endpoint (IND)	430	-0.092	1.2278	-0.009	-4.15	2.09	-0.078 (1.2137)	430	-0.014	0.0462	0.9586	0.000	-3.55	3.20
Week 3 (OP/MA)	946	0.003	1.2492	0.123	-5.81	2.09	-0.079 (1.2245)	937	0.087	0.0319	0.9760	0.000	-4.85	3.94
Week 16 (OP/MA)	907	-0.128	1.1783	-0.009	-3.67	2.10	-0.099 (1.2197)	901	-0.026	0.0318	0.9554	0.000	-2.69	3.45
Week 28 (OP/MA)	878	-0.019	1.2141	0.109	-3.51	4.40	-0.050 (1.1901)	872	0.036	0.0336	0.9922	0.000	-4.06	3.20
Week 40 (OP/MA)	848	-0.057	1.2104	0.109	-4.15	2.10	-0.066 (1.2048)	841	0.014	0.0328	0.9503	0.000	-3.07	3.45
Week 52 (OP/MA)	818	0.054	1.2022	0.217	-5.05	2.10	-0.070 (1.2170)	812	0.122	0.0353	1.0057	0.000	-3.69	3.20
Week 64 (OP/MA)	780	0.108	1.1880	0.310	-4.18	2.10	-0.068 (1.2146)	773	0.183	0.0343	0.9523	0.226	-3.45	3.45
Week 76 (OP/MA)	771	0.033	1.1872	0.123	-3.51	2.10	-0.073 (1.2085)	765	0.110	0.0357	0.9869	0.000	-3.78	3.69
Week 88 (OP/MA)	737	0.045	1.2158	0.345	-4.15	2.10	-0.070 (1.2128)	732	0.113	0.0377	1.0194	0.000	-3.61	3.69
Week 100 (OP/MA)	730	0.077	1.2238	0.345	-3.33	2.10	-0.076 (1.1995)	727	0.157	0.0373	1.0053	0.000	-2.96	3.94
Week 112 (OP/MA)	682	0.067	1.2108	0.303	-4.75	2.34	-0.065 (1.2042)	680	0.131	0.0401	1.0460	0.000	-4.29	3.94
Week 124 (OP/MA)	673	0.076	1.2021	0.303	-3.67	2.10	-0.072 (1.1899)	673	0.149	0.0407	1.0568	0.000	-4.26	3.94
Week 136 (OP/MA)	632	0.076	1.1920	0.123	-3.57	2.10	-0.083 (1.2105)	632	0.159	0.0441	1.1094	0.224	-3.20	4.19
Week 148 (OP/MA)	517	0.216	1.1330	0.369	-3.57	2.10	-0.049 (1.2198)	517	0.265	0.0465	1.0570	0.226	-2.48	3.94
Week 160 (OP/MA)	393	0.247	1.1666	0.369	-3.08	2.09	-0.052 (1.2089)	393	0.299	0.0551	1.0924	0.236	-3.20	3.69
Week 172 (OP/MA)	158	0.451	1.1161	0.616	-2.83	2.10	0.013 (1.2707)	158	0.439	0.0965	1.2126	0.246	-2.26	3.94
Week 184 (OP/MA)	52	0.388	1.1018	0.406	-2.83	2.10	0.191 (1.2251)	52	0.197	0.1581	1.1401	0.231	-2.36	2.84
Week 196 (OP/MA)	18	0.605	0.8694	0.862	-1.11	1.80	0.419 (1.1763)	18	0.186	0.2724	1.1556	-0.113	-1.23	2.96
Week 208 (OP/MA)	2	0.582	0.6687	0.582	0.11	1.05	1.409 (0.5015)	2	-0.827	0.1182	0.1672	-0.827	-0.95	-0.71
Week 220 (OP/MA)	1	1.291	-	1.291	1.29	1.29	1.054 (-)	1	0.236	-	-	0.236	0.24	0.24
Endpoint (OP/MA)	980	0.040	1.2658	0.303	-5.81	2.10	-0.076 (1.2267)	971	0.117	0.0359	1.1189	0.224	-4.85	3.94
HVLT, delayed recall														
Baseline (IND)	1007	-0.265	1.1832	-0.022	-5.46	1.30								
Day 28 (IND)	424	-0.099	2.1508	-0.022	-3.94	37.24	-0.264 (1.2021)	424	0.165	0.0980	2.0183	0.000	-2.60	36.98
Endpoint (IND)	424	-0.104	2.1547	-0.022	-3.94	37.24	-0.264 (1.2021)	424	0.160	0.0979	2.0150	0.000	-2.60	36.98
Week 3 (OP/MA)	946	-0.183	1.1836	0.039	-6.14	1.30	-0.265 (1.1801)	937	0.084	0.0287	0.8793	0.000	-3.23	3.80
Week 16 (OP/MA)	907	-0.205	1.8301	-0.022	-4.42	42.37	-0.277 (1.1757)	901	0.071	0.0557	1.6730	0.000	-3.26	41.30
Week 28 (OP/MA)	878	-0.196	1.1734	-0.022	-4.37	3.54	-0.227 (1.1425)	872	0.035	0.0314	0.9278	0.000	-3.80	3.80
Week 40 (OP/MA)	848	-0.252	1.2142	-0.022	-4.37	1.30	-0.255 (1.1625)	841	0.000	0.0332	0.9618	0.000	-3.92	3.80
Week 52 (OP/MA)	818	-0.133	1.4679	0.150	-4.42	24.43	-0.262 (1.1585)	812	0.128	0.0467	1.3318	0.000	-4.81	26.09
Week 64 (OP/MA)	780	-0.127	1.2020	0.316	-5.46	2.00	-0.257 (1.1476)	773	0.131	0.0348	0.9675	0.000	-5.98	4.41
Week 76 (OP/MA)	771	-0.149	1.1839	0.039	-4.42	1.30	-0.269 (1.1551)	765	0.120	0.0350	0.9685	0.000	-4.33	4.35
Week 88 (OP/MA)	737	-0.145	1.1976	0.260	-4.86	1.30	-0.262 (1.1534)	732	0.115	0.0371	1.0038	0.000	-4.41	5.43
Week 100 (OP/MA)	730	-0.149	1.1759	0.039	-3.94	1.30	-0.270 (1.1482)	727	0.125	0.0355	0.9585	0.000	-3.43	3.80

Task	N	Mean	SD	Median	Min	Max	Baseline Mean (SD)	Change from Baseline N	Mean*	SE	SD	Median*	Min	Max
Week 112 (OP/MA)	682	-0.132	1.1694	0.260	-3.91	1.32	-0.272 (1.1591)	680	0.138	0.0377	0.9840	0.000	-4.17	3.80
Week 124 (OP/MA)	672	-0.121	1.1417	0.039	-3.46	1.30	-0.267 (1.1412)	672	0.146	0.0386	1.0004	0.000	-3.85	3.80
Week 136 (OP/MA)	632	-0.193	1.2451	0.039	-4.91	1.32	-0.283 (1.1572)	632	0.090	0.0419	1.0545	0.000	-3.85	4.89
Week 148 (OP/MA)	517	0.014	1.0688	0.385	-3.83	1.32	-0.197 (1.1542)	517	0.211	0.0444	1.0099	0.000	-2.72	3.80
Week 160 (OP/MA)	393	-0.020	1.1578	0.385	-4.37	1.32	-0.205 (1.1724)	393	0.185	0.0495	0.9808	0.000	-3.26	3.80
Week 172 (OP/MA)	158	0.202	1.0144	0.522	-3.28	1.30	-0.133 (1.1941)	158	0.335	0.0824	1.0362	0.000	-2.72	3.80
Week 184 (OP/MA)	52	0.067	1.0506	0.212	-3.83	1.30	-0.040 (1.1411)	52	0.106	0.1582	1.1407	0.000	-4.89	2.17
Week 196 (OP/MA)	18	0.412	0.7259	0.522	-1.30	1.07	0.527 (0.7169)	18	-0.115	0.1961	0.8320	0.000	-2.60	1.09
Week 208 (OP/MA)	2	0.385	0.0000	0.385	0.38	0.38	0.865 (0.0000)	2	-0.481	0.0000	0.0000	-0.481	-0.48	-0.48
Week 220 (OP/MA)	1	0.865	-	0.865	0.87	0.87	0.865 (-)	1	0.000	-	-	0.000	0.00	0.00
Endpoint (OP/MA)	980	-0.142	1.2206	0.260	-6.14	1.32	-0.258 (1.1819)	971	0.114	0.0327	1.0189	0.000	-3.85	4.41

HVLT-R = Hopkins Verbal Learning Test-Revised; IND = induction phase; Max = maximum; Min = minimum; OP/MA = optimization/maintenance phase; SD = standard deviation; SE = standard error

* Positive values indicate improvement from baseline in cognitive performance; negative values indicate worsening.

Notes: Study baseline is applied. Detection = Simple Reaction Time test; Identification = Choice Reaction Time test.

Table S7. Mean Change from Study Baseline in Z-score Over Time Among Participants ≥ 65 Years Old (Induction and Optimization/Maintenance Phases of SUSTAIN-3)

Task	N	Mean	SD	Median	Min	Max	Baseline Mean (SD)	Change from Baseline N	Mean*	SE	SD	Median*	Min	Max
Detection														
Baseline (IND)	122	-0.734	1.3614	-0.525	-4.27	1.74								
Day 28 (IND)	13	-0.659	1.3127	-0.582	-3.04	1.56	-0.986 (1.8313)	13	0.327	0.3751	1.3523	-0.133	-1.76	2.84
Endpoint (IND)	13	-0.659	1.3127	-0.582	-3.04	1.56	-0.986 (1.8313)	13	0.327	0.3751	1.3523	-0.133	-1.76	2.84
Week 3 (OP/MA)	114	-0.679	1.4127	-0.581	-4.59	1.94	-0.694 (1.3696)	114	0.015	0.1091	1.1653	-0.063	-3.33	3.32
Week 16 (OP/MA)	104	-0.849	1.4497	-0.640	-4.62	1.58	-0.838 (1.3077)	104	-0.011	0.1194	1.2177	-0.051	-4.25	3.29
Week 28 (OP/MA)	98	-0.752	1.3918	-0.897	-5.50	1.90	-0.741 (1.3509)	98	-0.010	0.1162	1.1506	-0.055	-2.69	3.29
Week 40 (OP/MA)	95	-0.766	1.3873	-0.779	-5.34	1.65	-0.780 (1.2843)	95	0.014	0.1431	1.3943	0.115	-4.02	3.64
Week 52 (OP/MA)	90	-0.943	1.3552	-0.947	-4.77	1.69	-0.806 (1.3497)	90	-0.138	0.1304	1.2370	-0.128	-3.47	2.81
Week 64 (OP/MA)	87	-1.075	1.4885	-0.711	-5.32	1.71	-0.861 (1.2954)	87	-0.213	0.1424	1.3280	-0.221	-5.62	3.00
Week 76 (OP/MA)	90	-1.043	1.4773	-0.942	-5.62	1.60	-0.775 (1.3328)	90	-0.268	0.1286	1.2199	-0.186	-4.45	3.38
Week 88 (OP/MA)	85	-1.051	1.6062	-0.755	-5.72	1.85	-0.774 (1.2808)	85	-0.277	0.1477	1.3620	-0.148	-5.02	2.82
Week 100 (OP/MA)	78	-1.052	1.5394	-0.845	-5.90	1.56	-0.770 (1.2832)	78	-0.281	0.1480	1.3068	-0.024	-4.88	3.29
Week 112 (OP/MA)	78	-0.962	1.3194	-0.853	-4.58	1.96	-0.717 (1.2122)	78	-0.245	0.1283	1.1328	-0.189	-3.02	2.75
Week 124 (OP/MA)	73	-1.078	1.3708	-0.804	-4.75	1.39	-0.766 (1.2143)	73	-0.312	0.1546	1.3206	-0.168	-3.94	3.40
Week 136 (OP/MA)	54	-0.958	1.3815	-0.774	-4.18	1.60	-0.833 (1.2243)	54	-0.125	0.1744	1.2818	-0.152	-3.46	3.51
Week 148 (OP/MA)	64	-0.744	1.1797	-0.544	-3.79	1.35	-0.655 (1.0866)	64	-0.089	0.1379	1.1031	-0.108	-2.78	2.14
Week 160 (OP/MA)	63	-1.121	1.2891	-1.169	-5.00	1.44	-0.679 (1.1334)	63	-0.441	0.1530	1.2146	-0.388	-3.25	2.14
Week 172 (OP/MA)	17	-0.203	1.2539	0.008	-3.47	1.51	-0.561 (1.2171)	17	0.358	0.2637	1.0872	0.230	-1.98	3.17
Week 184 (OP/MA)	9	-0.540	1.2747	-0.306	-2.54	1.63	-1.052 (1.2341)	9	0.511	0.1695	0.5084	0.488	-0.55	1.24
Week 196 (OP/MA)	1	-0.001	-	-0.001	0.00	0.00	-0.186 (-)	1	0.185	-	-	0.185	0.19	0.19
Week 208 (OP/MA)	0	-	-	-	-	-	-	0	-	-	-	-	-	-
Week 220 (OP/MA)	0	-	-	-	-	-	-	0	-	-	-	-	-	-
Endpoint (OP/MA)	120	-0.939	1.4120	-0.890	-5.90	1.94	-0.744 (1.3699)	120	-0.195	0.1240	1.3579	-0.140	-3.25	4.02
Identification														
Baseline (IND)	122	-0.588	1.4468	-0.477	-5.83	2.35								
Day 28 (IND)	13	-0.802	1.8238	-0.292	-4.69	0.81	-0.916 (2.0912)	13	0.114	0.2498	0.9007	-0.124	-0.99	2.13
Endpoint (IND)	13	-0.802	1.8238	-0.292	-4.69	0.81	-0.916 (2.0912)	13	0.114	0.2498	0.9007	-0.124	-0.99	2.13
Week 3 (OP/MA)	114	-0.636	1.3802	-0.489	-5.16	2.15	-0.531 (1.3998)	114	-0.105	0.0984	1.0509	-0.053	-5.82	3.14
Week 16 (OP/MA)	106	-0.711	1.4037	-0.320	-5.28	1.54	-0.687 (1.4362)	106	-0.025	0.0947	0.9755	-0.109	-3.14	2.67
Week 28 (OP/MA)	100	-0.753	1.4508	-0.581	-6.82	2.57	-0.627 (1.4338)	100	-0.127	0.1105	1.1045	-0.192	-3.20	3.24
Week 40 (OP/MA)	96	-0.939	1.6268	-0.650	-5.75	1.62	-0.615 (1.4179)	96	-0.324	0.1329	1.3017	-0.327	-5.58	2.45
Week 52 (OP/MA)	90	-0.924	1.4540	-0.709	-6.26	2.27	-0.572 (1.5017)	90	-0.352	0.1429	1.3557	-0.418	-5.28	3.91
Week 64 (OP/MA)	88	-1.155	1.4770	-0.807	-6.40	1.19	-0.622 (1.4632)	88	-0.533	0.1381	1.2956	-0.419	-6.23	4.21
Week 76 (OP/MA)	89	-1.036	1.4591	-0.919	-6.40	1.49	-0.499 (1.3851)	89	-0.537	0.1370	1.2926	-0.337	-5.87	3.69
Week 88 (OP/MA)	86	-1.123	1.6989	-0.829	-6.91	1.38	-0.509 (1.2981)	86	-0.614	0.1547	1.4349	-0.637	-7.12	3.66

Task	N	Mean	SD	Median	Min	Max	Baseline Mean (SD)	Change from Baseline N	Mean*	SE	SD	Median*	Min	Max
Week 100 (OP/MA)	79	-1.004	1.6046	-0.683	-7.29	2.26	-0.479 (1.3156)	79	-0.525	0.1604	1.4256	-0.527	-7.63	4.05
Week 112 (OP/MA)	79	-1.130	1.4171	-1.011	-6.06	1.52	-0.484 (1.2552)	79	-0.646	0.1270	1.1291	-0.592	-4.16	1.26
Week 124 (OP/MA)	74	-1.186	1.6015	-1.033	-6.30	1.77	-0.495 (1.2648)	74	-0.691	0.1567	1.3476	-0.527	-5.86	1.97
Week 136 (OP/MA)	53	-1.060	1.4638	-0.826	-5.26	1.12	-0.541 (1.3503)	53	-0.519	0.1829	1.3313	-0.419	-6.64	2.07
Week 148 (OP/MA)	64	-0.946	1.2988	-0.703	-3.54	1.57	-0.337 (1.0458)	64	-0.609	0.1622	1.2976	-0.484	-5.36	2.12
Week 160 (OP/MA)	63	-1.012	1.2993	-0.846	-4.00	1.78	-0.327 (1.0731)	63	-0.685	0.1702	1.3506	-0.630	-4.96	2.47
Week 172 (OP/MA)	17	-0.214	1.0777	-0.067	-2.25	1.33	-0.039 (0.9628)	17	-0.176	0.2420	0.9976	-0.407	-1.64	2.01
Week 184 (OP/MA)	9	-0.516	1.0076	-0.523	-2.43	1.02	-0.268 (1.0593)	9	-0.248	0.3024	0.9071	-0.228	-1.70	0.69
Week 196 (OP/MA)	1	1.058	-	1.058	1.06	1.06	0.327 (-)	1	0.731	-	-	0.731	0.73	0.73
Week 208 (OP/MA)	0	-	-	-	-	-	-	0	-	-	-	-	-	-
Week 220 (OP/MA)	0	-	-	-	-	-	-	0	-	-	-	-	-	-
Endpoint (OP/MA)	120	-0.955	1.5544	-0.823	-7.29	1.86	-0.587 (1.4588)	120	-0.368	0.1240	1.3583	-0.370	-4.96	5.20
One Card Learning														
Baseline (IND)	121	-0.482	1.3275	-0.385	-3.45	3.45								
Day 28 (IND)	13	-0.471	1.3227	-0.628	-2.78	1.56	-0.772 (1.4349)	13	0.300	0.3068	1.1061	0.128	-1.17	2.22
Endpoint (IND)	13	-0.471	1.3227	-0.628	-2.78	1.56	-0.772 (1.4349)	13	0.300	0.3068	1.1061	0.128	-1.17	2.22
Week 3 (OP/MA)	114	-0.372	1.3543	-0.488	-2.63	3.24	-0.483 (1.2549)	114	0.111	0.0997	1.0642	0.236	-2.66	3.62
Week 16 (OP/MA)	107	-0.532	1.3490	-0.628	-3.55	3.99	-0.540 (1.2667)	106	0.027	0.1019	1.0492	-0.023	-2.67	3.07
Week 28 (OP/MA)	101	-0.304	1.4037	-0.262	-3.25	3.18	-0.473 (1.2715)	100	0.184	0.1185	1.1852	0.117	-3.05	2.74
Week 40 (OP/MA)	94	-0.380	1.3549	-0.427	-3.59	2.71	-0.460 (1.3110)	93	0.092	0.1087	1.0481	0.168	-3.43	2.49
Week 52 (OP/MA)	89	-0.279	1.4036	-0.225	-3.89	2.96	-0.524 (1.2485)	88	0.236	0.1345	1.2622	0.313	-2.83	3.25
Week 64 (OP/MA)	87	-0.124	1.3752	-0.137	-3.14	3.24	-0.446 (1.2965)	87	0.323	0.1085	1.0119	0.210	-2.22	2.11
Week 76 (OP/MA)	90	-0.117	1.5149	0.117	-3.83	2.49	-0.416 (1.3198)	90	0.299	0.1327	1.2590	0.484	-3.95	2.65
Week 88 (OP/MA)	87	-0.281	1.6626	-0.262	-5.74	3.99	-0.411 (1.3292)	87	0.130	0.1618	1.5088	0.137	-9.19	3.77
Week 100 (OP/MA)	80	-0.238	1.4805	-0.122	-5.57	2.96	-0.522 (1.2664)	80	0.285	0.1513	1.3535	0.521	-6.37	3.00
Week 112 (OP/MA)	79	-0.346	1.4144	-0.225	-4.34	2.96	-0.586 (1.2416)	79	0.240	0.1303	1.1580	0.324	-3.60	2.22
Week 124 (OP/MA)	74	-0.314	1.5621	-0.044	-6.41	2.71	-0.602 (1.1915)	74	0.288	0.1881	1.6185	0.388	-8.34	2.97
Week 136 (OP/MA)	54	-0.183	1.4441	-0.064	-2.59	3.18	-0.551 (1.1904)	54	0.367	0.1731	1.2718	0.353	-2.57	3.09
Week 148 (OP/MA)	64	0.014	1.5347	0.237	-3.39	2.71	-0.508 (1.1735)	64	0.521	0.1630	1.3041	0.412	-3.40	3.88
Week 160 (OP/MA)	62	-0.017	1.4206	-0.068	-3.16	2.71	-0.601 (1.1544)	62	0.583	0.1517	1.1947	0.652	-1.96	3.10
Week 172 (OP/MA)	18	0.104	1.5622	0.258	-2.47	3.24	-0.495 (1.3023)	18	0.600	0.2673	1.1341	0.597	-1.83	2.50
Week 184 (OP/MA)	9	0.319	1.1541	0.472	-2.25	1.55	0.049 (1.3099)	9	0.270	0.3714	1.1142	0.560	-2.01	1.68
Week 196 (OP/MA)	1	-0.137	-	-0.137	-0.14	-0.14	0.792 (-)	1	-0.930	-	-	-0.930	-0.93	-0.93
Week 208 (OP/MA)	0	-	-	-	-	-	-	0	-	-	-	-	-	-
Week 220 (OP/MA)	0	-	-	-	-	-	-	0	-	-	-	-	-	-
Endpoint (OP/MA)	120	-0.182	1.4437	-0.137	-3.55	3.24	-0.494 (1.3241)	119	0.306	0.1105	1.2058	0.213	-3.43	3.10
One-Back														
Baseline (IND)	121	-0.064	1.3853	-0.037	-4.01	2.99								
Day 28 (IND)	14	0.006	0.8449	0.067	-1.88	1.38	-0.163 (0.9994)	14	0.169	0.2916	1.0909	-0.114	-0.75	2.94
Endpoint (IND)	14	0.006	0.8449	0.067	-1.88	1.38	-0.163 (0.9994)	14	0.169	0.2916	1.0909	-0.114	-0.75	2.94

Task	N	Mean	SD	Median	Min	Max	Baseline Mean (SD)	Change from Baseline N	Mean*	SE	SD	Median*	Min	Max
Week 3 (OP/MA)	115	-0.010	1.4280	0.181	-5.73	2.72	-0.033 (1.4091)	115	0.023	0.0972	1.0429	0.064	-4.48	3.75
Week 16 (OP/MA)	108	-0.074	1.2974	0.124	-3.49	2.78	-0.084 (1.3569)	107	0.031	0.0916	0.9477	0.038	-3.68	3.12
Week 28 (OP/MA)	101	-0.086	1.2327	0.121	-3.36	2.17	-0.067 (1.2566)	100	0.001	0.0968	0.9678	-0.062	-3.26	3.69
Week 40 (OP/MA)	95	-0.113	1.1607	-0.060	-3.48	2.23	-0.027 (1.2305)	94	-0.079	0.0972	0.9419	-0.042	-3.78	2.19
Week 52 (OP/MA)	90	-0.127	1.3938	-0.050	-4.34	4.08	-0.004 (1.2763)	89	-0.112	0.1390	1.3116	-0.046	-3.41	5.80
Week 64 (OP/MA)	88	-0.225	1.4327	0.033	-4.61	2.43	-0.044 (1.3063)	88	-0.181	0.1225	1.1494	-0.168	-4.17	2.18
Week 76 (OP/MA)	89	-0.157	1.4382	-0.007	-5.31	2.55	0.050 (1.2591)	89	-0.207	0.1299	1.2251	-0.176	-5.27	2.06
Week 88 (OP/MA)	86	-0.164	1.3702	0.034	-4.67	2.45	-0.052 (1.2850)	86	-0.112	0.1299	1.2049	0.010	-3.67	3.35
Week 100 (OP/MA)	78	-0.073	1.3267	0.030	-4.86	2.95	-0.048 (1.3156)	78	-0.025	0.1360	1.2009	-0.041	-3.63	3.41
Week 112 (OP/MA)	78	-0.094	1.2464	-0.035	-3.45	2.77	-0.039 (1.2908)	78	-0.056	0.1310	1.1570	-0.141	-3.76	2.87
Week 124 (OP/MA)	74	-0.120	1.3356	0.065	-3.38	2.46	-0.026 (1.3008)	74	-0.094	0.1488	1.2800	0.041	-3.99	3.26
Week 136 (OP/MA)	54	0.030	1.2324	-0.077	-3.41	2.45	0.035 (1.3238)	54	-0.006	0.1627	1.1957	0.005	-4.16	3.39
Week 148 (OP/MA)	64	0.026	1.1715	0.061	-2.45	2.42	-0.002 (1.2869)	64	0.028	0.1552	1.2412	-0.057	-5.17	2.99
Week 160 (OP/MA)	63	-0.001	1.2184	0.085	-3.26	2.78	-0.035 (1.2834)	63	0.033	0.1600	1.2701	0.102	-4.27	3.28
Week 172 (OP/MA)	18	0.438	1.1389	0.729	-1.88	2.15	0.213 (1.4458)	18	0.224	0.2914	1.2364	0.004	-1.74	3.28
Week 184 (OP/MA)	9	0.517	1.0277	0.288	-0.47	2.51	0.352 (1.7280)	9	0.165	0.3292	0.9876	-0.134	-0.76	2.46
Week 196 (OP/MA)	1	2.100	-	2.100	2.10	2.10	2.994 (-)	1	-0.895	-	-	-0.895	-0.89	-0.89
Week 208 (OP/MA)	0	-	-	-	-	-	-	0	-	-	-	-	-	-
Week 220 (OP/MA)	0	-	-	-	-	-	-	0	-	-	-	-	-	-
Endpoint (OP/MA)	120	-0.046	1.3552	0.108	-4.86	2.67	-0.060 (1.3968)	119	0.022	0.1085	1.1840	0.012	-4.27	3.28
Groton Maze Learning Test														
Baseline (IND)	115	0.154	1.0818	0.360	-4.90	1.83								
Day 28 (IND)	13	0.050	0.9971	0.398	-2.07	1.18	0.348 (1.0475)	13	-0.298	0.1436	0.5179	-0.217	-1.34	0.69
Endpoint (IND)	13	0.050	0.9971	0.398	-2.07	1.18	0.348 (1.0475)	13	-0.298	0.1436	0.5179	-0.217	-1.34	0.69
Week 3 (OP/MA)	107	0.326	0.8983	0.360	-3.26	2.05	0.200 (1.0927)	106	0.130	0.0818	0.8418	0.057	-2.78	2.90
Week 16 (OP/MA)	94	0.304	1.0317	0.494	-4.45	1.83	0.330 (0.8680)	93	-0.022	0.0812	0.7835	0.076	-4.68	1.53
Week 28 (OP/MA)	91	0.338	1.1342	0.589	-6.20	1.70	0.252 (1.0580)	90	0.086	0.0999	0.9476	0.087	-4.65	3.43
Week 40 (OP/MA)	87	0.244	0.8962	0.398	-2.41	1.74	0.269 (1.0334)	85	-0.024	0.0808	0.7447	-0.038	-3.03	3.24
Week 52 (OP/MA)	83	0.333	0.9261	0.551	-3.76	1.96	0.258 (1.0851)	81	0.108	0.0878	0.7901	0.087	-2.21	2.63
Week 64 (OP/MA)	79	0.301	0.9628	0.489	-2.53	1.87	0.218 (1.1346)	78	0.095	0.0966	0.8535	0.125	-2.77	3.55
Week 76 (OP/MA)	80	0.537	0.9029	0.665	-3.36	2.00	0.265 (1.0919)	79	0.273	0.0997	0.8859	0.303	-3.60	3.47
Week 88 (OP/MA)	82	0.345	1.0936	0.662	-4.02	2.05	0.220 (1.0743)	80	0.149	0.1110	0.9929	0.122	-3.05	3.81
Week 100 (OP/MA)	75	0.248	0.9266	0.446	-2.93	1.61	0.151 (1.1363)	74	0.100	0.1003	0.8625	0.081	-1.75	3.97
Week 112 (OP/MA)	70	0.411	0.8722	0.563	-1.66	1.92	0.217 (1.1183)	69	0.206	0.1045	0.8683	0.173	-1.87	3.24
Week 124 (OP/MA)	68	0.268	1.0140	0.522	-3.53	1.74	0.124 (1.1827)	67	0.160	0.1253	1.0255	0.173	-1.91	4.12
Week 136 (OP/MA)	50	0.369	1.0406	0.554	-3.58	1.66	0.112 (1.2835)	49	0.257	0.1519	1.0632	0.260	-2.04	3.42
Week 148 (OP/MA)	59	0.370	0.8856	0.474	-2.93	2.00	0.083 (1.1776)	58	0.306	0.1187	0.9038	0.133	-1.39	3.97
Week 160 (OP/MA)	55	0.440	0.8491	0.589	-1.59	1.74	0.082 (1.1931)	54	0.361	0.1271	0.9339	0.152	-1.45	3.32
Week 172 (OP/MA)	18	0.463	0.8937	0.665	-2.07	1.47	0.031 (1.1924)	17	0.429	0.1974	0.8139	0.381	-0.56	2.60
Week 184 (OP/MA)	9	-0.018	2.2755	0.589	-5.88	1.44	0.470 (0.8880)	9	-0.488	0.5091	1.5274	-0.130	-4.33	0.82

Task	N	Mean	SD	Median	Min	Max	Baseline Mean (SD)	Change from Baseline N	Mean*	SE	SD	Median*	Min	Max
Week 196 (OP/MA)	1	1.269	-	1.269	1.27	1.27	1.355 (-)	1	-0.087	-	-	-0.087	-0.09	-0.09
Week 208 (OP/MA)	0	-	-	-	-	-	-	0	-	-	-	-	-	-
Week 220 (OP/MA)	0	-	-	-	-	-	-	0	-	-	-	-	-	-
Endpoint (OP/MA)	116	0.270	1.0526	0.513	-5.88	1.74	0.160 (1.0841)	112	0.144	0.0911	0.9638	0.041	-4.33	3.32
HVLT-R, Word recall														
Baseline (IND)	122	-0.072	1.2771	-0.088	-3.96	2.45								
Day 28 (IND)	14	-0.722	1.2305	-0.818	-2.61	1.87	-0.660 (1.1606)	14	-0.062	0.2617	0.9792	0.208	-3.05	0.67
Endpoint (IND)	14	-0.722	1.2305	-0.818	-2.61	1.87	-0.660 (1.1606)	14	-0.062	0.2617	0.9792	0.208	-3.05	0.67
Week 3 (OP/MA)	117	0.017	1.3181	0.078	-3.06	2.45	-0.100 (1.2807)	117	0.117	0.0940	1.0172	0.000	-2.24	4.26
Week 16 (OP/MA)	110	-0.206	1.1912	-0.296	-3.28	2.26	-0.093 (1.2876)	110	-0.113	0.1004	1.0526	0.000	-2.47	3.59
Week 28 (OP/MA)	102	-0.111	1.1613	-0.031	-2.84	2.26	-0.095 (1.3094)	102	-0.016	0.1003	1.0127	0.000	-3.05	2.69
Week 40 (OP/MA)	97	-0.118	1.1330	-0.031	-2.61	2.26	-0.112 (1.3097)	97	-0.006	0.1133	1.1161	0.000	-2.86	4.04
Week 52 (OP/MA)	92	0.078	1.2528	0.078	-3.51	2.45	-0.174 (1.3220)	92	0.252	0.1139	1.0924	0.191	-2.29	3.36
Week 64 (OP/MA)	88	0.423	1.8566	0.303	-2.16	14.20	-0.137 (1.3123)	88	0.561	0.1949	1.8279	0.417	-2.29	14.35
Week 76 (OP/MA)	92	0.130	1.1077	0.231	-2.16	2.26	-0.043 (1.2899)	92	0.172	0.1150	1.1028	0.208	-1.91	3.63
Week 88 (OP/MA)	87	0.099	1.2161	0.078	-2.61	2.10	-0.077 (1.2985)	87	0.176	0.1271	1.1851	0.224	-2.29	3.81
Week 100 (OP/MA)	80	-0.081	1.1983	-0.146	-3.73	2.45	-0.121 (1.2707)	80	0.040	0.1325	1.1854	0.000	-2.10	4.71
Week 112 (OP/MA)	78	0.117	1.1738	0.303	-2.61	2.45	-0.141 (1.2757)	78	0.258	0.1253	1.1067	0.095	-1.91	3.81
Week 124 (OP/MA)	76	0.085	1.2318	0.119	-2.39	2.16	-0.150 (1.2880)	76	0.235	0.1244	1.0847	0.303	-1.91	3.59
Week 136 (OP/MA)	57	-0.140	1.1323	-0.221	-3.06	1.88	-0.133 (1.1422)	57	-0.007	0.1529	1.1540	-0.191	-3.36	2.48
Week 148 (OP/MA)	65	-0.040	1.2274	0.078	-3.06	2.10	-0.194 (1.2954)	65	0.154	0.1427	1.1504	0.224	-2.69	3.36
Week 160 (OP/MA)	63	-0.067	1.1385	0.160	-3.51	2.26	-0.246 (1.2725)	63	0.178	0.1462	1.1603	-0.191	-2.02	3.36
Week 172 (OP/MA)	18	0.417	1.2839	0.415	-1.94	2.45	-0.016 (1.1836)	18	0.433	0.2741	1.1630	0.208	-1.12	2.47
Week 184 (OP/MA)	9	0.807	0.8367	0.542	-0.15	2.26	0.290 (1.4070)	9	0.517	0.4250	1.2751	0.191	-0.95	2.47
Week 196 (OP/MA)	1	-0.818	-	-0.818	-0.82	-0.82	0.751 (-)	1	-1.570	-	-	-1.570	-1.57	-1.57
Week 208 (OP/MA)	0	-	-	-	-	-	-	0	-	-	-	-	-	-
Week 220 (OP/MA)	0	-	-	-	-	-	-	0	-	-	-	-	-	-
Endpoint (OP/MA)	120	0.022	1.3160	0.160	-3.51	2.26	-0.086 (1.2803)	120	0.108	0.1076	1.1786	0.000	-2.91	3.36
HVLT, delayed recall														
Baseline (IND)	122	-0.294	1.2347	-0.260	-3.39	1.39								
Day 28 (IND)	14	-0.456	1.0503	-0.260	-1.94	1.30	-0.510 (1.2455)	14	0.054	0.2426	0.9078	0.000	-1.48	1.56
Endpoint (IND)	14	-0.456	1.0503	-0.260	-1.94	1.30	-0.510 (1.2455)	14	0.054	0.2426	0.9078	0.000	-1.48	1.56
Week 3 (OP/MA)	117	-0.331	1.3277	-0.260	-3.91	1.39	-0.328 (1.2390)	117	-0.003	0.0791	0.8553	0.000	-2.08	2.60
Week 16 (OP/MA)	109	-0.358	1.2047	-0.463	-3.91	1.39	-0.325 (1.2452)	109	-0.033	0.1010	1.0550	0.000	-3.13	2.96
Week 28 (OP/MA)	102	-0.175	2.1371	-0.260	-2.86	17.97	-0.323 (1.2320)	102	0.148	0.1899	1.9184	0.000	-3.13	16.67
Week 40 (OP/MA)	96	-0.262	1.1708	-0.260	-3.06	1.54	-0.304 (1.2083)	96	0.042	0.1047	1.0262	0.000	-2.60	3.65
Week 52 (OP/MA)	92	-0.183	1.1478	-0.093	-2.34	1.39	-0.324 (1.2620)	92	0.141	0.1056	1.0129	0.000	-1.56	3.13
Week 64 (OP/MA)	88	0.021	1.1151	0.269	-2.86	1.39	-0.299 (1.2238)	88	0.320	0.1141	1.0702	0.000	-1.56	4.17
Week 76 (OP/MA)	92	-0.107	1.1503	0.260	-2.86	1.39	-0.241 (1.2049)	92	0.134	0.1077	1.0331	0.000	-3.65	3.65
Week 88 (OP/MA)	87	0.001	1.1073	0.260	-2.86	1.54	-0.278 (1.2109)	87	0.279	0.1208	1.1268	0.000	-1.56	4.69

Task	N	Mean	SD	Median	Min	Max	Baseline Mean (SD)	Change from Baseline N	Mean*	SE	SD	Median*	Min	Max
Week 100 (OP/MA)	80	-0.138	1.0904	-0.093	-2.86	1.39	-0.320 (1.1863)	80	0.182	0.1349	1.2065	0.000	-2.60	4.69
Week 112 (OP/MA)	78	-0.072	1.2116	0.260	-2.86	1.39	-0.340 (1.1902)	78	0.268	0.1320	1.1657	0.000	-2.22	3.65
Week 124 (OP/MA)	76	-0.082	1.2182	0.260	-4.43	1.39	-0.357 (1.1942)	76	0.274	0.1361	1.1863	0.000	-3.13	4.17
Week 136 (OP/MA)	57	-0.043	1.1023	-0.093	-2.34	1.54	-0.318 (1.1187)	57	0.276	0.1525	1.1516	0.000	-1.56	3.65
Week 148 (OP/MA)	65	-0.114	1.2516	-0.093	-3.91	1.39	-0.374 (1.1931)	65	0.261	0.1601	1.2906	0.000	-3.13	4.17
Week 160 (OP/MA)	63	-0.173	1.0329	-0.093	-3.39	1.39	-0.408 (1.1931)	63	0.234	0.1559	1.2378	0.000	-2.60	3.65
Week 172 (OP/MA)	18	0.179	1.4444	0.454	-4.43	1.39	-0.197 (1.3033)	18	0.376	0.2772	1.1760	0.521	-3.13	2.08
Week 184 (OP/MA)	9	0.433	0.9254	0.781	-1.30	1.30	0.366 (1.1520)	9	0.067	0.1611	0.4832	0.000	-0.74	0.52
Week 196 (OP/MA)	1	-0.781	-	-0.781	-0.78	-0.78	0.781 (-)	1	-1.563	-	-	-1.563	-1.56	-1.56
Week 208 (OP/MA)	0	-	-	-	-	-	-	0	-	-	-	-	-	-
Week 220 (OP/MA)	0	-	-	-	-	-	-	0	-	-	-	-	-	-
Endpoint (OP/MA)	120	-0.210	1.2644	-0.093	-4.43	1.39	-0.312 (1.2352)	120	0.102	0.1050	1.1500	0.000	-3.13	3.65

HVLT-R = Hopkins Verbal Learning Test-Revised; IND = induction phase; Max = maximum; Min = minimum; OP/MA = optimization/maintenance phase; SD = standard deviation; SE = standard error

* Positive values indicate improvement from baseline in cognitive performance; negative values indicate worsening.

Notes: Study baseline is applied. Detection = Simple Reaction Time test; Identification = Choice Reaction Time test.

Table S8. Most Severe Postbaseline Potentially Suicide-related Category vs. Baseline Based on Columbia-Suicide Severity Rating Scale (C-SSRS) Induction and Optimization/Maintenance Phases

	Most Severe Post Baseline Potentially Suicide-Related Category			
	No Event	Suicidal Ideation	Suicidal Behavior	Total
Baseline:				
No event	569 (49.7%)	49 (4.3%)	1 (0.1%)	619 (54.1%)
Suicidal ideation	160 (14.0%)	168 (14.7%)	9 (0.8%)	337 (29.5%)
Suicidal behavior	73 (6.4%)	109 (9.5%)	6 (0.5%)	188 (16.4%)
Total	802 (70.1%)	326 (28.5%)	16 (1.4%)	1144 (100.0%)

Note: Each participant is counted only once in the table, based on the most severe postbaseline C-SSRS category: No event = 0; suicidal ideation = 1,2,3,4,5; suicidal behavior = 6,7,8,9,10.
Note: Baseline assessed for lifetime is used.

Figure S3. Group Mean (± SE) Standardized (z-score) Change from Baseline for Psychomotor Function (Speed of Performance on the Detection Test) in SUSTAIN-3

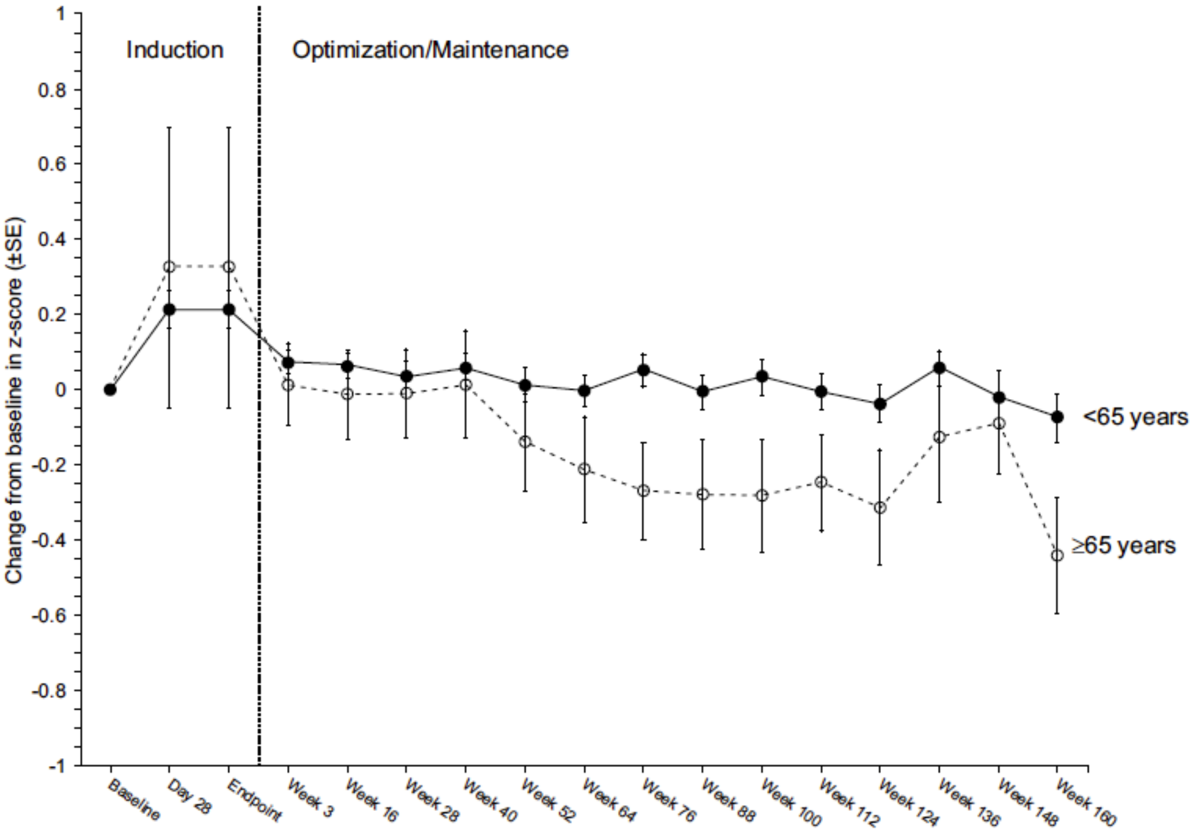

n <65 years	408	408	917	865	852	824	800	762	752	722	713	670	662	617	509	386
n ≥65 years	13	13	114	104	98	95	90	87	90	85	78	78	73	54	64	63

Detection test (Simple Reaction Time). Higher score indicates faster reaction time.

Figure S4. Group Mean (± SE) Standardized (z-score) Change from Baseline for Attention Function (Speed of Performance on the Identification Test) in SUSTAIN-3

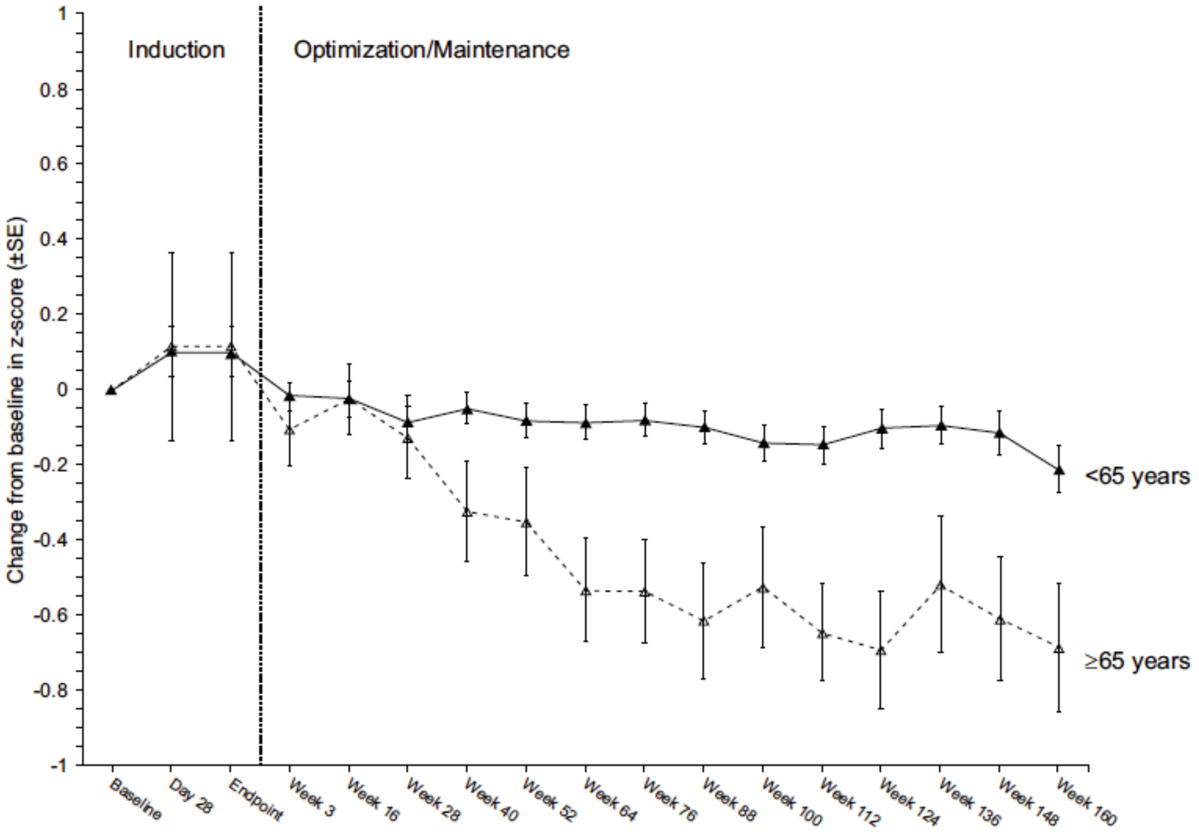

n <65 years	410	410	921	874	860	831	804	767	753	725	716	674	666	622	511	386
n ≥65 years	13	13	114	104	98	95	90	87	90	85	78	78	73	54	64	63

Identification test (Choice Reaction Time). Higher score indicates faster reaction time.

Figure S5. Mean (± SE) Patient Health Questionnaire (PHQ) Total Score (Observed Cases)

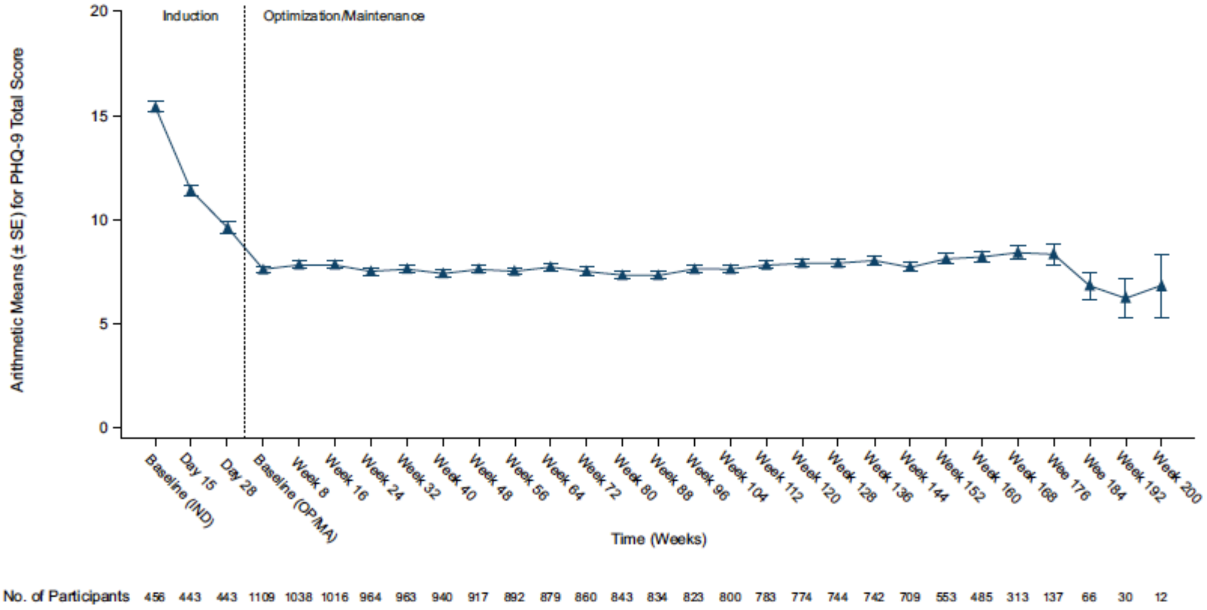

Note: Every-8-week data are presented. The visits with fewer than 10 participants are not presented.

Figure S6. Remission Based on Sheehan Disability Scale (SDS) Score Over Time (Observed Cases)

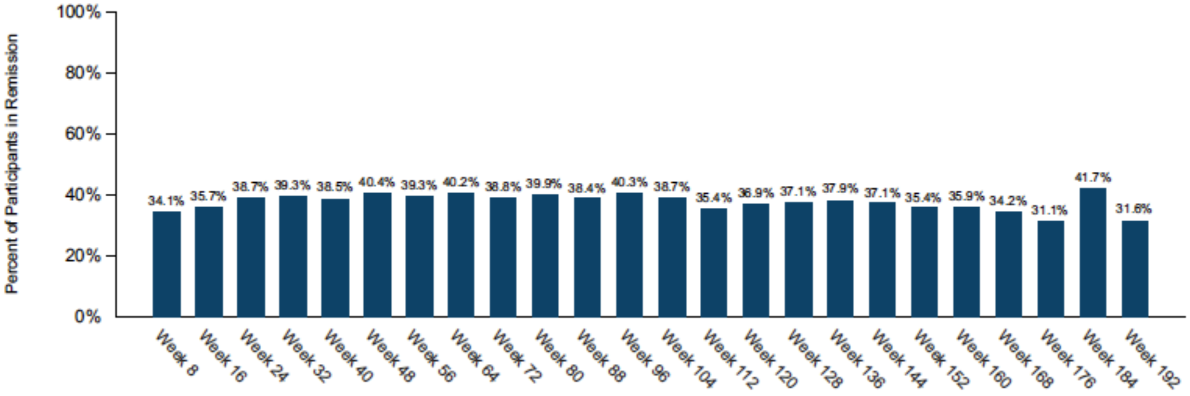

Participants in Remission	306	312	315	322	306	317	296	298	279	280	272	282	263	233	242	229	228	212	160	140	83	33	20	6
Total Participants	898	875	814	820	794	785	753	741	719	702	709	700	679	659	656	618	601	572	452	390	243	106	48	19

Note: Every-8-week data are presented. The visits with fewer than 10 participants are not presented. Remission is defined as SDS ≤2 for each item score and ≤6 for the total score.
